# Metal catalyst-free N-allylation/alkylation of imidazole and benzimidazole with Morita–Baylis–Hillman (MBH) alcohols and acetates

**DOI:** 10.3762/bjoc.19.93

**Published:** 2023-09-01

**Authors:** Olfa Mhasni, Jalloul Bouajila, Farhat Rezgui

**Affiliations:** 1 Laboratory of Materials, Treatement and Analysis LMTA, LR 15 INRAP 03, National Institute of Research and Physico-Chemical Analysis (INRAP), Biotechpôle Sidi Thabet 2020, Tunisia; 2 Paul Sabatier University, Toulouse 3, Laboratoire de Génie Chimique UMR 5503 Toulouse, France; 3 University of Tunis El Manar, Laboratory of Organic Chemistry, Faculty of Sciences, Campus, 2092 Tunis, Tunisia

**Keywords:** allylic substitution, aza-Michael addition, imidazole, Morita–Baylis–Hillman

## Abstract

A highly α-regioselective N-nucleophilic allylic substitution of cyclic MBH alcohols and acetates with imidazole or benzimidazole, in toluene at reflux with an azeotropic distillation, was successfully carried out with no catalysts or additives, affording the corresponding N-substituted imidazole derivatives in good yields. On the other hand, in refluxing toluene or methanol, the aza-Michael addition of imidazole onto acyclic MBH alcohols was performed using DABCO as an additive, leading to the corresponding 1,4-adducts in 70–84% yields.

## Introduction

Morita–Baylis–Hillman (MBH) adducts are multifunctionalized compounds having both a hydroxy moiety and a Michael acceptor unit. They have found application as valuable synthons and useful precursors for the synthesis of various biologically active molecules [1–3]. Recently, MBH adducts, as electrophilic substrates, have been employed to achieve fruitful results in allylic substitution reactions with various nucleophiles, including C- and heteronucleophiles, such as compounds bearing –OH, –SH, and –NH groups [4–7]. Among them, the carbon–nitrogen bond formation through N-nucleophilic substitution reactions plays a central role for the synthesis of numerous compounds exhibiting various biological activities [1–7].

**Figure 1:**
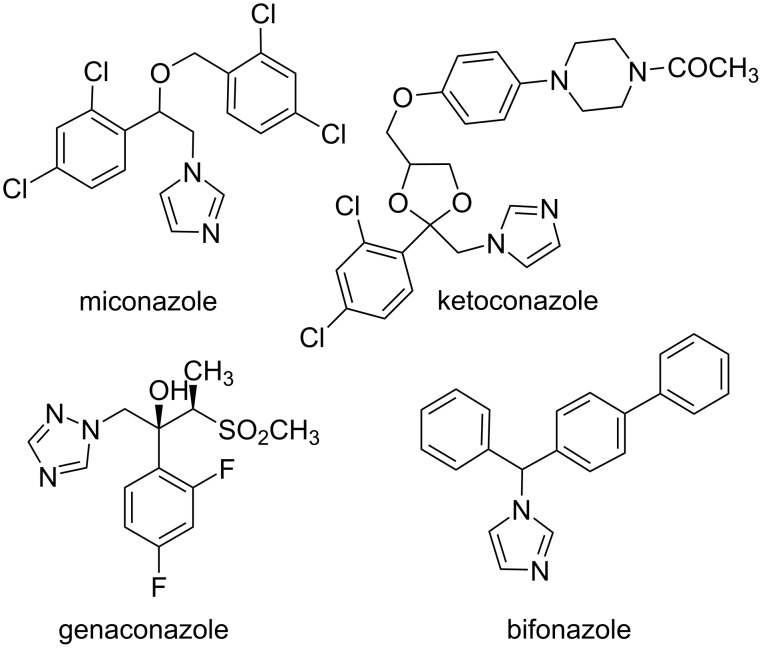
Medicines containing an imidazole nucleus.

In this context, the imidazole moiety is widely known as one of the most important groups which plays efficient roles in bioactive compounds [8]. For instance, a number of N-substituted imidazole derivatives, such as miconazole, ketoconazole, genaconazole, and bifonazole have become well-established drugs for the treatment of numerous mycotic infections (Figure 1) [9–10]. Therefore, the development of new methods for the preparation of such compounds is highly required.

The MBH acetates, instead of the corresponding alcohols, have been extensively used as precursors in nucleophilic allylic substitution reactions with amines, presumably due to the perceived poor leaving group ability and low reactivity of the hydroxy group. Interestingly, the direct nucleophilic substitution of the corresponding alcohols has drawn much attention because of the availability of these substrates and the formation of water as the sole non-toxic byproduct in the reaction [11]. In general, the previous methods for the amination of MBH alcohols needed catalysts or additives such as FeCl_3_ [12–13], In(OTf)_3_ [14], MoCl_5_ [15], AuCl_3_ [16], and I_2_ [17] as Lewis acids. Alternatively, Yang et al. [18–19] have developed a catalytic system involving Pd/Ti(OiPr)_4_ or Pd/carboxylic acid for the direct allylation of anilines with alcohols.

**Scheme 1:**
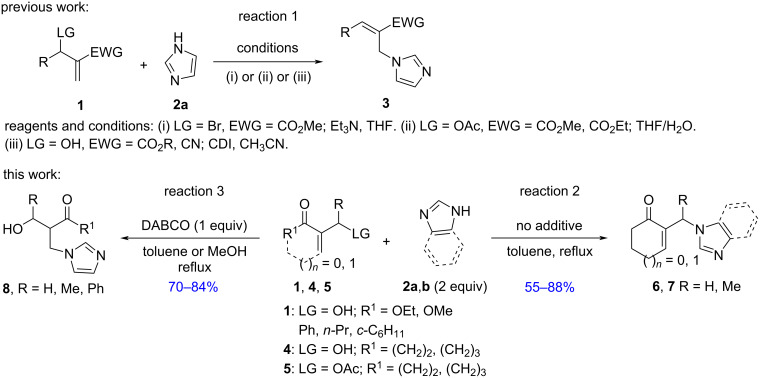
Synthesis of N-substituted imidazole derivatives from MBH adducts.

The synthesis of *N*-allylimidazole derivatives **3** has been previously carried out using acyclic MBH adducts bearing good leaving groups, such as bromide derivatives in Et_3_N/THF mixtures [20] (Scheme 1, reaction 1, i) and acetates in THF/water [21] (Scheme 1, reaction 1, ii), or MBH alcohols in the presence of CDI (1,1’-carbonyldiimidazole) in acetonitrile (Scheme 1, reaction 1, iii) [22]. In the last case, as the hydroxy moiety is not a good leaving group, such alcohols were in situ converted into the corresponding *O*-allyl carbamates as leaving groups, followed by their reaction with imidazoles, affording the S_N_2’ products **3** (Scheme 1, reaction 1, iii).

Correlatively, we have previously reported a direct amination of cyclic MBH alcohols **4** with morpholine in the presence of imidazole (**2a**), as a powerful nucleophilic additive, affording, via competitive allylic nucleophilic substitution in toluene at reflux, a mixture of the corresponding N-substituted morpholine and N-substituted imidazole derivatives **6** [23]. In addition, a literature survey showed that nucleophilic allylic substitution reactions of acyclic/cyclic MBH adducts **1**, **4**, or **5**, bearing good or poor leaving groups, using imidazole derivatives as nucleophilic reagents, have not been extensively developed. Therefore, in continuation of our previous study on the nucleophilic allylic substitution of MBH adducts [23–26], we disclose in this work a simple and efficient procedure for the synthesis of N-substituted imidazoles **6**–**8**. The products were obtained either through direct conversions of the corresponding cyclic MBH alcohols **4** as well as acetates **5** in the presence of imidazoles **2a** and **2b** as nucleophilic reagents without catalysts or activating agents (Scheme 1, reaction 2), or from acyclic MBH alcohols **1** using DABCO as a powerful nucleophilic additive (Scheme 1, reaction 3).

## Results and Discussion

In our first investigations, we selected the reaction of the primary acetate **5a** [27] as the model substrate bearing a good leaving group, with imidazole (**2a**, 2 equiv) as a powerful nucleophilic reagent. The reaction was achieved with no need of a catalyst or any additive in toluene at reflux affording within 24 h the S_N_2-type product **6a** in 82% yield (Table 1, entry 1). Similarly, the five-membered acetate **5b** reacted under the same conditions and gave the *N*-allyl-substituted imidazole **6b** in 65% yield (Table 1, entry 2).

**Table 1:** Allylation of imidazole derivatives **2a**,**b** with cyclic MBH adducts **4** and **5**.

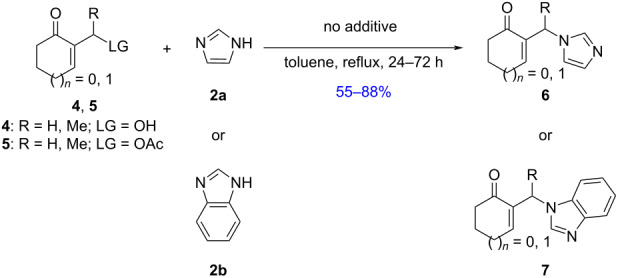					

Entry	MBH adduct **4** or **5**	Imidazole **2a** or **2b**	Time (h)	Product **6** or **7**	Yield (%) **6** or **7**

1	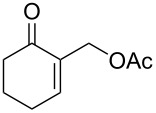 **5a**	**2a**	24	**6a**	82
2	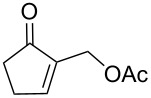 **5b**	**2a**	24	**6b**	65
3	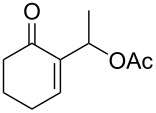 **5c**	**2a**	24	**6c**	75
4	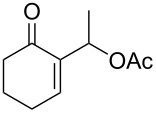 **5c**	**2b**	48	**7a**	87
5	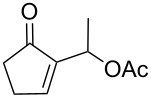 **5d**	**2a**	24	**6d**	69
6	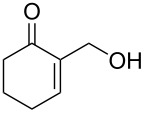 **4a**	**2a**	48	**6a**	88
7	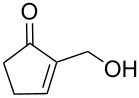 **4b**	**2a**	24	**6b**	55
8	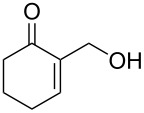 **4a**	**2b**	72	**7b**	80
9	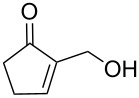 **4b**	**2b**	72	**7c**	72
10	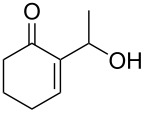 **4c**	**2a**	48	**6c**	76
11	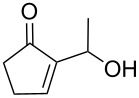 **4d**	**2a**	24	**6d**	60
12	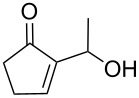 **4d**	**2b**	72	**7d**	85

Furthermore, treatment of the available secondary acetates **5c**,**d** (R = Me) with imidazoles **2a** and **2b** (2 equiv) in refluxing toluene afforded the *N*-substituted imidazoles **6c**, **6d**, and **7a** within ca. 24 h in 69–87% yields (Table 1, entries 3–5).

Having established the optimized conditions for the amination of primary and secondary acetates **5a–d** (Table 1, entries 1–5), carrying a good leaving group (OAc), we turned our attention to the investigation of the direct amination of MBH alcohols **4a–d**, with a poor leaving group (OH). Under the previous conditions (2 equiv of imidazole, toluene, reflux), the conversion of alcohol **4a** [28] into the corresponding imidazole **6a** was very slow and the starting materials were almost recovered. However, the continuous removal of water formed from the direct amination of alcohol **4a** by azeotropic distillation shifted the position of the equilibrium in direction to the formation of the allylated imidazole **6a** which was obtained in good 88% yield (Table 1, entry 6). The protocol was also successfully extended to the reaction of the primary five-membered alcohol **4b** [29] with imidazole (**2a**) as well as to that of alcohols **4a**,**b** with benzimidazole (**2b**), leading to the S_N_2-type products **6b** and **7b**,**c**, respectively, in 55–80% yields (Table 1, entries 7–9).

In addition, we have shown that the direct amination of the available secondary alcohols **4c**,**d** (R = Me) [30] could be achieved with imidazole derivatives **2a**,**b** under the conditions established above affording within 24–72 h the allylation products **6c**,**d** and **7d** in 60–85% yields (Table 1, entries 10–12).

**Scheme 2:**
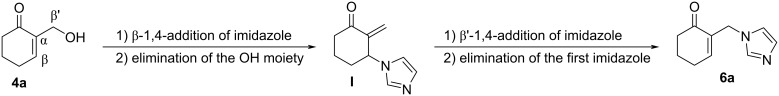
Proposed mechanism for the allylation of imidazole with alcohol **4a**.

Mechanistically, we believe that the nucleophilic allylic substitutions of alcohols **4**, such as **4a**, starts with a conjugate addition of imidazole (**2a**) at the C-β position of the Michael acceptor **4a**, followed by elimination of the hydroxy moiety, affording the intermediate **I**. Similarly, a further second β’-conjugate addition of imidazole (**2a**) to **I** might occur, followed by elimination of imidazole (**2a**) finally providing the allylated derivative **6a** (Scheme 2) [24–26,31]. It is notable, that such reaction mechanism involving the intermediate **I** was previously explored by Smith [32] and supported by studies of Tamura [33].

Next, in order to explore the scope of the above process, we have also investigated the direct allylation of imidazole (**2a**) with acyclic MBH alcohol **1a**. In our first experiment, this substrate did not react with imidazole (**2a**) in toluene at reflux within 24 h, with or without azeotropic distillation, and the starting materials were completely recovered (Table 2, entry 1). Moreover, the addition of additives to the previous reaction mixture, such as DMAP [24–26,31] or molecular sieves 4 Å, commonly used to mediate nucleophilic allylic substitutions, did not lead to a notable improvement of the reaction outcome (Table 2, entries 2 and 3). However, the use of DABCO, commonly used as a powerful catalyst or a nucleophilic additive in the reaction of acyclic MBH adducts with various nucleophiles [21,34–37], did not afford the S_N_2/S_N_2’ products but provided the 1,4-adduct **8a** in 84% yield (Table 2, entry 4).

**Table 2:** Optimization of the reaction conditions of imidazole (**2a**) with acyclic MBH **1a**.

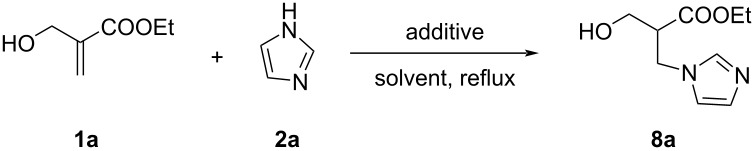				

Entry	Additive (1 equiv)	Solvent	Time (h)	**8a**, yield (%)

1	none	toluene	24	n.r.
2	MS 4 Å	toluene	24	n.r.
3	DMAP	toluene	24	25
4	DABCO	toluene	24	84
5	none	MeOH	10	65
6	DABCO	MeOH	10	68

Alternatively, we also investigated the reaction of alcohol **1a** [38] and imidazole (**2a**, 2 equiv), without any catalyst or additive in refluxing methanol, a solvent commonly employed in the conversion of MBH adducts using a variety of amines [39–40]. Our study showed that the imidazole (**2a**) reacted with alcohol **1a**, without any additive or in the presence of DABCO as additive, in a 1,4-fashion leading to the imidazole derivative **8a**, within 10 h in 65–68% yield (Table 2, entries 5 and 6).

Therefore, in our further experiments on the imidazole-mediated conversion of acyclic MBH alcohols, toluene at reflux was retained as the solvent of choice for the reaction using DABCO as additive.

Next, the treatment of acrylate-derived alcohols **1b**,**c** (**1b**, EWG = CO_2_Et, R = Ph; **1c**, EWG = CO_2_Me, R = Me) [41], under the previously optimized conditions afforded the corresponding 1,4-adducts **8b**,**c** in 70–75% yield (Table 3, entries 2 and 3), as 55:45 and 59:41 mixtures of inseparable diastereomers, respectively. The relative diastereomeric ratios (dr) were determined by means of ^1^H NMR based on the proton at the α-position of the EWG moiety (Table 3).

**Table 3:** Michael addition of imidazole (**2a**) onto acyclic MBH alcohols **1a**–**f**.

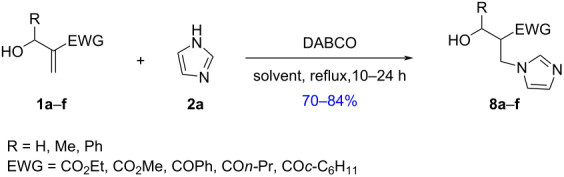						

Entry	Product	R	EWG	Solvent	Time (h)	**8**, yield (%), dr^a^

1	**8a**	H	CO_2_Et	toluene	24	84, none
2	**8b**	Ph	CO_2_Et	toluene	24	75, 55:45
3	**8c**	Me	CO_2_Me	toluene	24	70, 59:41
4	**8d**	H	COPh	MeOH	10	70, none
5	**8e**	H	CO*n*-Pr	MeOH	12	76, none
6	**8f**	H	COC_6_H_11_	MeOH	12	73, none

^a^Determined by ^1^H NMR spectroscopy from the crude reaction mixture.

In order to explore the scope of this synthetic approach, we have studied the reaction of ketone-derived alcohols such as **1d** (EWG = COPh, R = H), **1e** (EWG = CO*n*-Pr, R = H), **1f** (EWG = CO*c*-C_6_H_11_, R = H) [42], and imidazole under the established reaction conditions and we have observed that the conversion was complete but was not clean. However, in methanol at reflux a clean reaction took place providing the corresponding 1,4-adducts **8d–f** in 70–76% yields (Table 3, entries 4–6).

## Conclusion

We have successfully developed an efficient N-nucleophilic allylic substitution protocol of cyclic MBH alcohols **4** and acetates **5** with imidazoles in refluxing toluene. The new N-substituted imidazoles **6** and **7** were afforded in high purity and good yields. In toluene or methanol at reflux temperature, acyclic MBH alcohols reacted with imidazole in a 1,4-fashion, leading to the corresponding Michael adducts **8** in 70–84% yields.

Synthetic applications of such imidazole derivatives [22,43–44], as well as their biological evaluation [45–47] are underway in our laboratory.

## Experimental

### Typical procedure for the α-substitution of cyclic MBH adducts with imidazoles

A mixture of allyl acetate **5a** (2 mmol, 0.33 g) or allyl alcohol **4a** (2 mmol, 0.25 g) and imidazole (**2a**, 4 mmol, 0.27 g) in toluene (25 mL) was heated under reflux (for **5a**) or in a Dean–Stark apparatus (for **4a**). After completion (TLC), the reaction mixture was cooled, washed with brine, and dried. The toluene was removed and the residue was purified by column chromatography on silica gel (acetone/ether 8:2) to give the pure N-substituted imidazole **6a**.

### Typical procedure for the preparation of imidazole derivatives **8**

A mixture of acyclic MBH alcohol **1** (1 mmol), imidazole (**2a**, 2 mmol) and DABCO (1 mmol), was stirred at reflux temperature of methanol or toluene. After completion of the reaction, the solvent was removed by rotary evaporation and CH_2_Cl_2_ (10 mL) was added. The mixture was washed with brine and dried. Finally, the solvent was removed and the residue was purified by column chromatography on silica gel, using acetone/ether as eluent, to give the pure imidazole derivative **8**.

## Supporting Information

File 1 Full experimental details and characterization data of all new compounds.

File 2 ^1^H and ^13^C NMR and HRMS spectra of compounds.
