# Supplementary material for: Metal catalyst-free N-allylation/alkylation of imidazole and benzimidazole with Morita–Baylis–Hillman (MBH) alcohols and acetates
Source: Beilstein J Org Chem. 2023 Sep 1;19:1251–8. doi: 10.3762/bjoc.19.93 (PMC10477972; doi:10.3762/bjoc.19.93)
Supplement: File 1 — Full experimental details and characterization data of all new compounds. [file Beilstein_J_Org_Chem-19-1251-s001.pdf]

BEILSTEIN JOURNAL OF ORGANIC CHEMISTRY

Supporting Information

for

Metal catalyst-free N-allylation/alkylation of imidazole and benzimidazole with Morita–Baylis–Hillman (MBH) alcohols and acetates

Olfa Mhasni, Jalloul Bouajila and Farhat Rezgui

Beilstein J. Org. Chem. **2023,** *19,* 1251–1258. doi:10.3762/bjoc.19.93

Full experimental details and characterization data of all new compounds

License and Terms: This is a supporting information file under the terms of the Creative Commons Attribution License (https://creativecommons.org/licenses/by/4.0). Please note that the reuse, redistribution and reproduction in particular requires that the author(s) and source are credited and that individual graphics may be subject to special legal provisions.
The license is subject to the *Beilstein Journal of Organic Chemistry* terms and conditions: (https://www.beilstein-journals.org/bjoc/terms)

Experimental section

All cyclic MBH alcohols **4** [1-3], acetates **5** [4], and acyclic MBH alcohols **1a**,**f** [5-7] were obtained as previously described. IR spectra were recorded on a Bruker (IFS 66v/S) spectrometer. ^{1}H NMR and ^{13}C NMR spectra were recorded either on a Bruker AC-500 spectrometer (500 MHz for ^{1}H and 125 MHz for ^{13}C) or an AC-300 spectrometer (300 MHz for ^{1}H and 75 MHz for ^{13}C) in $CDCl_3$, using TMS as an internal standard (chemical shifts in δ values, *J* in Hz). High resolution mass spectra (HRMS) were recorded as EI-HRMS on an Autospec Ultima/micromass mass spectrometer. Analytical thin layer chromatography (TLC) was performed using Fluka Kieselgel 60 F254 pre-coated silica gel plates. Visualization was achieved by UV light (254 nm). Flash chromatography was performed using Merck silica gel 60 and a gradient solvent system ether/acetone as eluent.

Typical procedure for the α-substitution of cyclic MBH adducts with imidazoles

A mixture of allyl acetate **5a** (2 mmol, 0.33 g) or allyl alcohol **4a** (2 mmol, 0.25 g) and imidazole (**2a**, 4 mmol, 0.27 g) in toluene (25 mL) was heated under reflux (for **5a**) or in a Dean stark apparatus (for **4a**). After completion (TLC), the reaction mixture was cooled, washed with brine, and dried. The toluene was removed and the residue was purified by column chromatography on silica gel (acetone/ether 8:2) to give the pure N-substituted imidazole **6a**.

2-((1*H*-Imidazol-1-yl)methyl)cyclohex-2-enone (6a) [8]

Yield: 82%; yellow oil; ν ($CHCl_3$) 2932, 1666, 1503, 1380, 1227, 1074 cm^{-1}; ^{1}H NMR ($CDCl_3$, 500 MHz): δ_H 7.47 (s, 1H), 7.01 (s, 1H), 6.89 (s, 1H), 6.64 (t, *J* = 4.0 Hz, 1H), 4.70 (s, 2H), 2.47–2.35 (m, 4H), 2.04–1.98 (m, 2H); ^{13}C NMR ($CDCl_3$, 125 MHz): δ_C 197.6, 147.7, 137.6, 135.2, 129.3, 119.4, 45.5, 37.9, 25.8, 22.6; HRMS (EI): MH^+, found 177.1022. $C_{10}H_{13}N_2O$ requires 177.1028.

2-((1*H*-Imidazol-1-yl)methyl)cyclopent-2-enone (6b)

Yield: 65%; yellow oil; ν ($CHCl_3$) 2924, 1693, 1504, 1388, 1227, 1073 cm^{-1}; ^{1}H NMR ($CDCl_3$, 500 MHz): δ_H 7.39 (s, 1H), 7.34 (t, *J* = 4.0 Hz, 1H), 7.02 (s, 1H), 6.94 (s, 1H), 4.71 (s, 2H), 2.64–2.60 (m, 2H), 2.46–2.43 (m, 2H); ^{13}C NMR ($CDCl_3$, 125 MHz): δ_C 207.2, 160.2, 142.1, 137.4, 129.5, 119.3, 41.7, 34.6, 26.7; HRMS (EI): MH^+, found 163.0866. $C_9H_{11}N_2O$ requires 163.0871.

2-((1*H*-Imidazol-1-yl)ethyl)cyclohex-2-enone (6c)

Yield: 75%; yellow oil; ν ($CHCl_3$) 2935, 1665, 1497, 1381, 1226, 1077 cm^{-1}; ^{1}H NMR ($CDCl_3$, 500 MHz): δ_H 7.55 (s, 1H), 7.03 (s, 1H), 6.93 (s, 1H), 6.57 (t, *J* = 4.0 Hz, 1H), 5.34 (q, *J* = 6.2 Hz, 1H), 2.47–2.36 (m, 4H), 2.02–1.96 (m, 2H), 1.62 (d, *J* = 6.2 Hz, 3H) ; ^{13}C NMR ($CDCl_3$, 125 MHz): δ_C 197.3, 145.4, 140.4, 136.2, 128.9, 117.8, 50.4, 38.4, 25.7, 22.4, 19.8; MS (m/z): 191 (13), 190 (M+, 100), 189 (8), 175 (15), 162 (16), 149 (7), 134 (7), 123 (62), 107 (10), 95 (48), 81 (27), 79 (38), 69 (50), 67 (75), 55 (50); HRMS (EI): MH^+, found 191.1188. $C_{11}H_{15}N_2O$ requires 191.1184.

2-((1*H*-Imidazol-1-yl)ethyl)cyclopent-2-enone (6d)

Yield: 69%; yellow oil; ν ($CHCl_3$) 2926, 1695, 1496, 1395, 1227, 1077 cm^{-1}; ^{1}H NMR ($CDCl_3$, 500 MHz): δ_H 7.59 (s, 1H), 7.28 (t, *J* = 4.0 Hz, 1H), 7.03 (s, 1H), 7.01 (s, 1H), 5.11 (q, *J* = 6.0 Hz, 1H), 2.62–2.60 (m, 2H), 2.46–2.43 (m, 2H), 1.71 (d, *J* = 6.0 Hz, 3H) ; ^{13}C NMR ($CDCl_3$, 125 MHz): δ_C 207.2, 159.1, 146.6, 136.0, 129.0, 117.8, 49.1, 35.2, 26.6, 19.5; MS (m/z): 177 (12), 176 (M+, 100), 175 (7), 161 (1), 147 (9), 109 (43), 81 (54), 79 (74), 69 (33), 67 (13), 53 (31); HRMS (EI): MH^+, found 177.1031. $C_{10}H_{13}N_2O$ requires 177.1028.

2-((1*H*-Benzimidazol-1-yl)ethyl)cyclohex-2-enone (7a)

Yield: 87%; yellow oil; ^{1}H NMR ($CDCl_3$, 500 MHz): δ_H 8.03 (s, 1H), 7.73–7.71 (m, 1H), 7.21–7.15 (m, 3H), 6.49 (t, *J* = 4.0 Hz, 1H), 5.52 (q, *J* = 6.0 Hz, 1H), 2.38–2.34 (m, 2H), 2.25–2.19 (m, 2H), 1.89–1.83 (m, 2H), 1.70 (d, *J* = 6.0 Hz, 3H); ^{13}C NMR ($CDCl_3$, 125 MHz): δ_C 197.5, 146.0, 143.7, 141.3, 133.9, 133.2, 122.8, 122.1, 120.1, 110.4, 49.1, 38.1, 25.6, 22.3, 19.4 ; MS (m/z): 241 (17), 240 (M+, 100), 239 (10), 226 (15), 225 (100), 211 (10), 197 (7), 183 (5), 169 (5), 145 (14), 119 (15), 118 (55), 91 (8), 77 (7), 67 (9), 55 (5); HRMS (EI): MH^+, found 241.1347. $C_{15}H_{17}N_2O$ requires 241.1341.

2-((1*H*-Benzimidazol-1-yl)methyl)cyclohex-2-enone (7b)

Yield: 80%; yellow oil; ^{1}H NMR ($CDCl_3$, 500 MHz): δ_H 7.85 (s, 1H), 7.68–7.62 (m, 1H), 7.22–7.08 (m, 3H), 6.49 (t, *J* = 4.0 Hz, 1H), 4.78 (s, 2H), 2.30–2.25 (m, 2H), 2.15–2.10 (m, 2H), 1.82–1.74 (m, 2H); ^{13}C NMR ($CDCl_3$, 125 MHz): δ_C 197.7, 147.8, 143.6, 143.3, 133.8, 133.3, 122.6, 121.7, 119.9, 109.6, 43.2, 37.6, 25.3, 22.2; MS (m/z): 227 (16), 226 (M+, 100), 225 (33), 211 (5), 198 (28), 197 (19), 183 (7), 170 (30), 169 (24), 157 (7), 131 (22), 118 (15), 104 (4), 90 (5), 77 (7), 63 (3), 53 (6); HRMS (EI): MH^+, found 227.1189. $C_{14}H_{15}N_2O$ requires 227.1184.

2-((1*H*-Benzimidazol-1-yl)methyl)cyclopent-2-enone (7c)

Yield: 72%; yellow oil; ^{1}H NMR ($CDCl_3$, 500 MHz): δ_H 7.92 (s, 1H), 7.75–7.74 (m, 1H), 7.29–7.18 (m, 4H), 4.87 (s, 2H), 2.51–2.49 (m, 2H), 2.39–2.37 (m, 2H); ^{13}C NMR ($CDCl_3$, 125 MHz): δ_C 207.6, 160.6, 143.6, 143.4, 140.9, 133.4, 123.1, 122.2, 120.3, 109.6, 39.7, 34.5, 26.7; MS (m/z): 213 (14), 212 (M+, 100), 211 (46), 197 (2), 184 (11), 183 (39), 169 (17), 156 (21), 131 (10), 118 (14), 104 (5), 90 (4), 77 (5), 63 (3), 53 (3); HRMS (EI): MH^+, found 213.1033. $C_{13}H_{13}N_2O$ requires 213.1028.

2-((1*H*-Benzimidazol-1-yl)ethyl)cyclopent-2-enone (7d)

Yield: 85%; yellow oil; ^{1}H NMR ($CDCl_3$, 500 MHz): δ_H 8.01 (s, 1H), 7.72–7.69 (m, 1H), 7.25–7.12 (m, 4H), 5.25 (q, *J* = 6.0 Hz, 1H), 2.46–2.42 (m, 2H), 2.31–2.30 (m, 2H), 1.74 (d, *J* = 6.0 Hz, 3H) ; ^{13}C NMR ($CDCl_3$, 125 MHz): δ_C 207.1, 159.3, 145.1, 143.4, 141.1, 132.9, 122.7, 122.1, 120.02, 110.2, 47.4, 34.8, 26.2, 18.6; MS (m/z): 227 (16), 226 (M+, 100), 225 (8), 211 (38), 197 (14), 183 (13), 169 (4), 156 (1), 119 (12), 118 (74), 109 (8), 91 (9), 79 (14),77 (7), 63 (5), 53 (7); HRMS (EI): MH^+, found 227.1190. $C_{14}H_{15}N_2O$ requires 227.1184.

Typical procedure for the preparation of imidazole derivatives 8

A mixture of acyclic MBH alcohol **1** (1 mmol), imidazole (**2a**, 2 mmol) and DABCO (1 mmol), was stirred at reflux temperature of methanol or toluene. After completion of the reaction, the solvent was removed by rotary evaporation and CH_2Cl_2 (10 mL) was added. The mixture was washed with brine and dried. Finally, the solvent was removed and the residue was purified by a column chromatography on silica gel, using acetone/ether as eluent, to give the pure imidazole derivative **8**.

Ethyl 2-((1*H*-imidazol-1-yl)methyl)-3-hydroxypropanoate (8a)

Yield: 84%; yellow oil; ν ($CHCl_3$) 3118, 2982, 2934, 1723, 1509, 1451, 1376, 1282, 1225, 1181, 1071 cm^{-1}; ^{1}H NMR ($CDCl_3$, 300 MHz): δ_H 7.52 (s, 1H), 6.99 (s, 1H), 6.95 (s, 1H), 4.77 (s, 1H, O<u>H</u>), 4.35 (d, *J* = 6.6 Hz, 2H), 4.14 (q, *J* = 7.1 Hz, 2H), 3.81–3.73 (m, 2H), 2.97–2.91 (m, 1H), 1.22 (t, *J* = 7.1 Hz, 3H); ^{13}C NMR ($CDCl_3$, 75 MHz): δ_C 171.8, 137.8, 128.7, 119.6, 61.2, 59.3, 49.2, 44.6, 14.0; HRMS (EI): MH^+, found 199.1085. $C_9H_{15}N_2O_3$ requires 199.1083.

Ethyl 2-((1*H*-imidazol-1-yl)methyl)-3-hydroxy-3-phenylpropanoate (8b)

Overall yield: 75%; yellow oil; dr = 55:45. Major diastereomer ^{1}H NMR ($CDCl_3$, 300 MHz): δ_H 7.41–7.22 (m, 6H), 6.83–6.76 (m, 2H), 4.85 (d, *J* = 6.7 Hz, 1H), 4.46–3.81 (m, 4H), 3.19–3.12 (m, 1H), 1.06 (t, *J* = 7.1 Hz, 3H); ^{13}C NMR ($CDCl_3$, 75 MHz): δ_C 172.0, 141.1, 137.3, 129.0, 128.2, 128.2, 126.1, 119.1, 72.6, 61.2, 55.0, 45.6, 13.9. Minor diastereomer ^{1}H NMR ($CDCl_3$, 300 MHz): δ_H 7.41–7.22 (m, 6H), 6.83–6.76 (m, 2H), 4.92 (d, *J* = 7.3 Hz, 1H), 4.46–3.81 (m, 4H), 3.07–3.01 (m, 1H), 0.91 (t, *J* = 7.1 Hz, 3H); ^{13}C NMR ($CDCl_3$, 75 MHz): δ_C 171.8, 141.7, 137.4, 129.0, 128.4, 128.0, 126.3, 119.3, 72.6, 61.., 55.8, 45.3, 13.7; HRMS (EI): MH^+, found 275.1402. $C_{15}H_{19}N_2O_3$ requires 275.1396.

Methyl 2-((1*H*-imidazol-1-yl)methyl)-3-hydroxybutanoate (8c)

Overall yield: 70%; yellow oil; dr = 59:41. Major diastereomer ^{1}H NMR ($CDCl_3$, 300 MHz): δ_H δ_H 7.46 (s, 1H), 6.98 (s, 1H), 6.89 (s, 1H), 5.17 (s, 1H, O<u>H</u>), 4.43–4.30 (m, 2H), 4.05–3.99 (m, 1H), 3.62 (s, 3H), 2.84–2.77 (m, 1H), 1.27 (d, *J* = 6.2 Hz, 3H); ^{13}C NMR ($CDCl_3$, 75 MHz): δ_C 172.8, 137.4, 128.8, 119.4, 66.1, 55.5, 52.0, 45.9, 21.9. Minor diastereomer ^{1}H NMR ($CDCl_3$, 300 MHz): δ_H 7.49 (s, 1H), 6.99 (s, 1H), 6.93 (s, 1H), 5.17 (s, 1H, O<u>H</u>), 4.43–4.30 (m, 2H), 4.05–3.99 (m, 1H), 3.67 (s, 3H), 2.92–2.87 (m, 1H), 1.25 (d, *J* = 6.4 Hz, 3H); ^{13}C NMR ($CDCl_3$, 75 MHz): δ_C 172.3, 137.5, 128.9, 119.4, 65.8, 54.3, 52.0, 45.0, 20.7; HRMS (EI): MH^+, found 199.1085. $C_9H_{15}N_2O_3$ requires 199.1083.

3-Hydroxy-2-((1*H*-imidazol-1-yl)methyl)-1-phenylpropan-1-one (8d)

Yield: 70%; ^{1}H NMR ($CDCl_3$, 300 MHz): δ_H 7.85–7.82 (m, 2H), 7.56–7.36 (m, 4H), 6.90 (s, 1H), 6.87 (s, 1H), 6.41 (s, 1H, O<u>H</u>), 4.52–4.34 (m, 2H), 4.02–3.94 (m, 1H), 3.88–3.70 (m, 2H); ^{13}C NMR ($CDCl_3$, 75 MHz): δ_C 199.8, 137.6, 136.1,

133.6, 128.8, 128.7, 128.2, 119.7, 60.7, 51.5, 45.1; HRMS (EI): MH^+, found 231.1132. $C_{13}H_{15}N_2O_2$ requires 231.1134.

1-Hydroxy-2-((1*H*-imidazol-1-yl)methyl)hexan-3-one (8e)

Yield: 76%; ^{1}H NMR ($CDCl_3$, 300 MHz): δ_H 7.45 (s, 1H), 6.97 (s, 1H), 6.89 (s, 1H), 4.63 (s, 1H, O<u>H</u>), 4.36–4.17 (m, 2H), 3.80–3.69 (m, 2H), 3.11–3.05 (m, 1H), 2.56–2.26 (m, 2H), 1.56–1.49 (m, 2H), 0.84 (t, J = 7.3 Hz, 3H); ^{13}C NMR ($CDCl_3$, 75 MHz): δ_C 210.6, 137.7, 128.9, 119.5, 59.8, 55.7, 44.9, 44.5, 16.7, 13.5; HRMS (EI): MH^+, found 197.1293. $C_{10}H_{17}N_2O_2$ requires 197.1290.

1-Cyclohexyl-3-hydroxy-2-((1*H*-imidazol-1-yl)methyl)propan-1-one (8f)

Yield: 73%; ^{1}H NMR ($CDCl_3$, 300 MHz): δ_H 7.43 (s, 1H), 6.96 (s, 1H), 6.88 (s, 1H), 4.71 (s, 1H, O<u>H</u>), 4.36–4.16 (m, 2H), 3.74–3.41 (m, 2H), 3.31–3.24 (m, 1H), 2.42–2.34 (m, 1H), 1.72–1.54 (m, 5H), 1.43–1.15 (m, 5H); ^{13}C NMR ($CDCl_3$, 75 MHz): δ_C 213.6, 137.6, 128.8, 119.5, 60.3, 54.4, 50.8, 45.0, 29.6, 28.0, 27.2, 25.7, 25.2; HRMS (EI): MH^+, found 237.1607. $C_{13}H_{21}N_2O_2$ requires 237.1603.

References

1. Rezgui, F.; El Gaïed, M. M. *Tetrahedron Lett.* **1998**, *39*, 5965.

2. Gatri, R.; El Gaïed, M. M. *Tetrahedron Lett.* **2002**, *43*, 7835.

3. Luo, S.; Wang, P. G.; Cheng, J. P. *J. Org. Chem.* **2004**, *69*, 555.

4. Rezgui, F.; El Gaied, M. M. *Tetrahedron* **1997,** *53*, 15711.

5. Villieras, J.; Rambaud, M. *Org. Synth. Coll. Vol. VIII*, **1993**, 265.

6. Yu, C.; Luo, B.; Hu, L. *J. Org. Chem.* **2001**, *66*, 5413.

7. Ben Kraïem, J.; Ben Ayed, T.; Amri, H. *Tetrahedron Lett.* **2006**, *47*, 7077.

8. Oueslati, Y.; Baioui, N.; Rezgui, F. *Synth. Commun.* **2017**, *47*, 892.
