# Supplementary material for: Metal catalyst-free N-allylation/alkylation of imidazole and benzimidazole with Morita–Baylis–Hillman (MBH) alcohols and acetates
Source: Beilstein J Org Chem. 2023 Sep 1;19:1251–8. doi: 10.3762/bjoc.19.93 (PMC10477972; doi:10.3762/bjoc.19.93)
Supplement: File 2 — 1H and 13C NMR and HRMS spectra of compounds. [file Beilstein_J_Org_Chem-19-1251-s002.pdf]

BEILSTEIN JOURNAL OF ORGANIC CHEMISTRY

Supporting Information

for

Metal catalyst-free N-allylation/alkylation of imidazole and benzimidazole with Morita–Baylis–Hillman (MBH) alcohols and acetates

Olfa Mhasni, Jalloul Bouajila and Farhat Rezgui

Beilstein J. Org. Chem. **2023,** *19,* 1251–1258. doi:10.3762/bjoc.19.93

^{1}H and ^{13}C NMR and HRMS spectra of compounds

License and Terms: This is a supporting information file under the terms of the Creative Commons Attribution License (https://creativecommons.org/licenses/by/4.0). Please note that the reuse, redistribution and reproduction in particular requires that the author(s) and source are credited and that individual graphics may be subject to special legal provisions.
The license is subject to the *Beilstein Journal of Organic Chemistry* terms and conditions: (https://www.beilstein-journals.org/bjoc/terms)

Table of contents

^{1}H NMR, ^{13}C NMR, and dept 135 NMR spectra S2–S21

ESI-HRMS spectra S22–S35

$^{1}H/^{13}C$ NMR spectra for compounds 6a–d, 7a–d, 8a–f

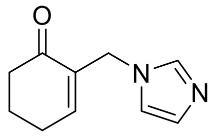

6a

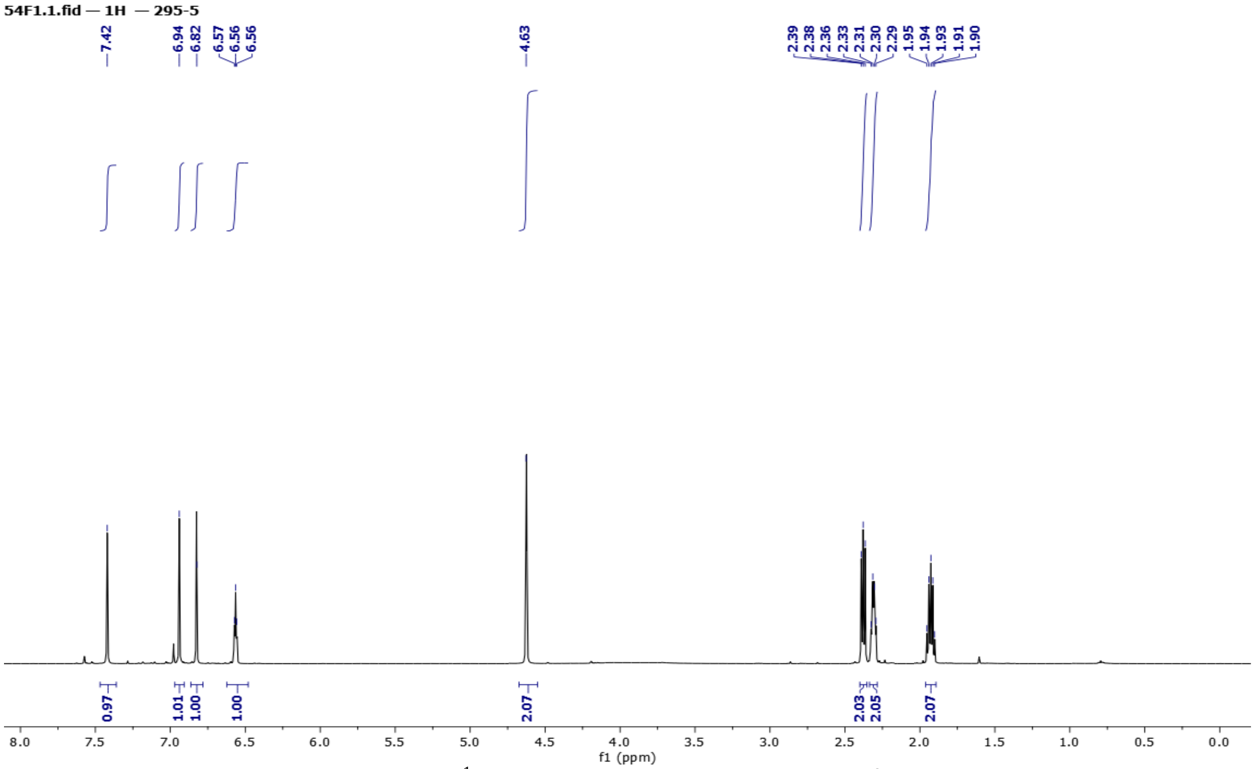

Figure S1. ^{1}H NMR (500 MHz, $CDCl_3$) spectra of **6a**

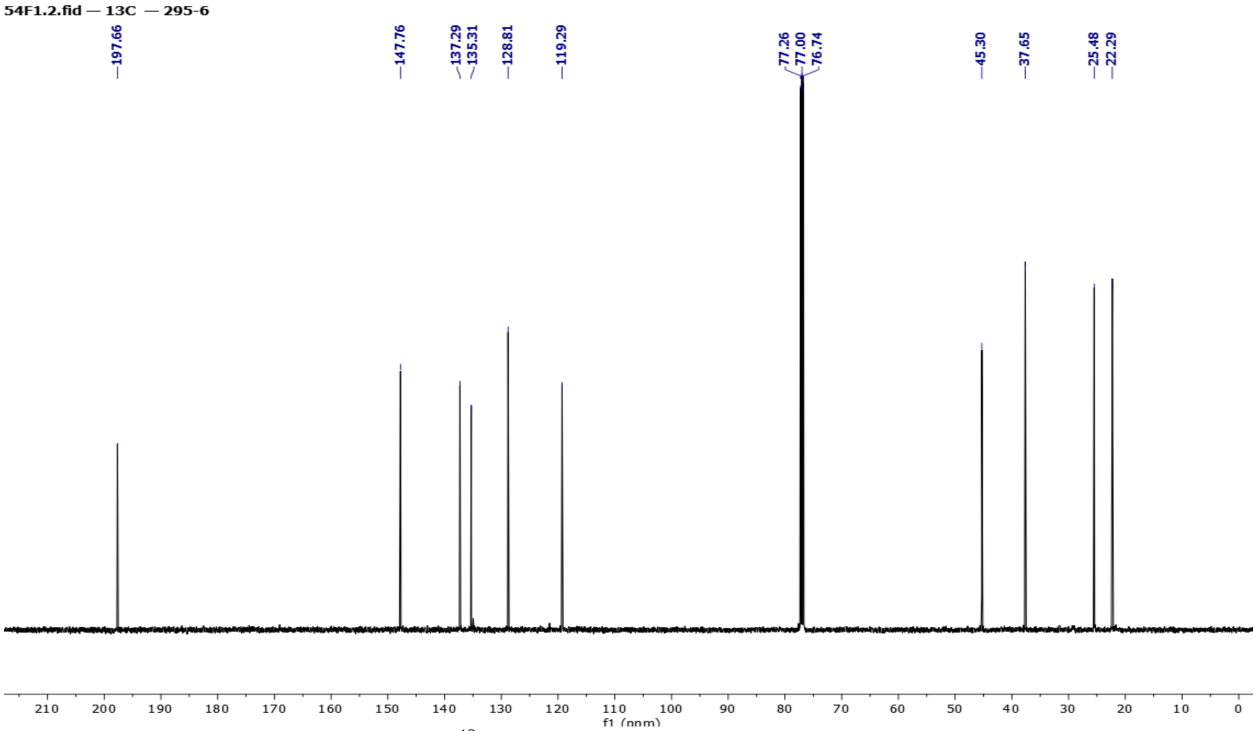

Figure S2. ^{13}C NMR (125 MHz, $CDCl_3$) spectra of **6a**

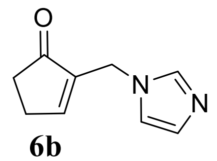

6b

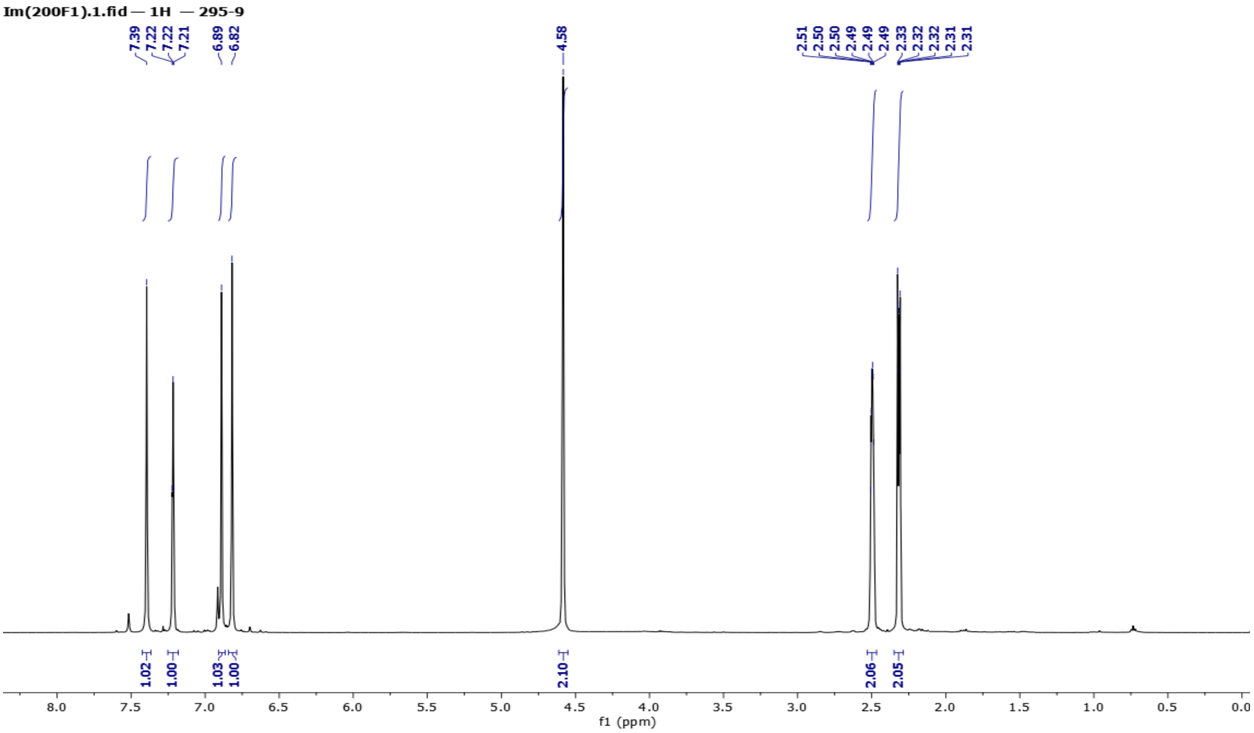

Figure S3. ^{1}H NMR (500 MHz, $CDCl_3$) spectra of **6b**

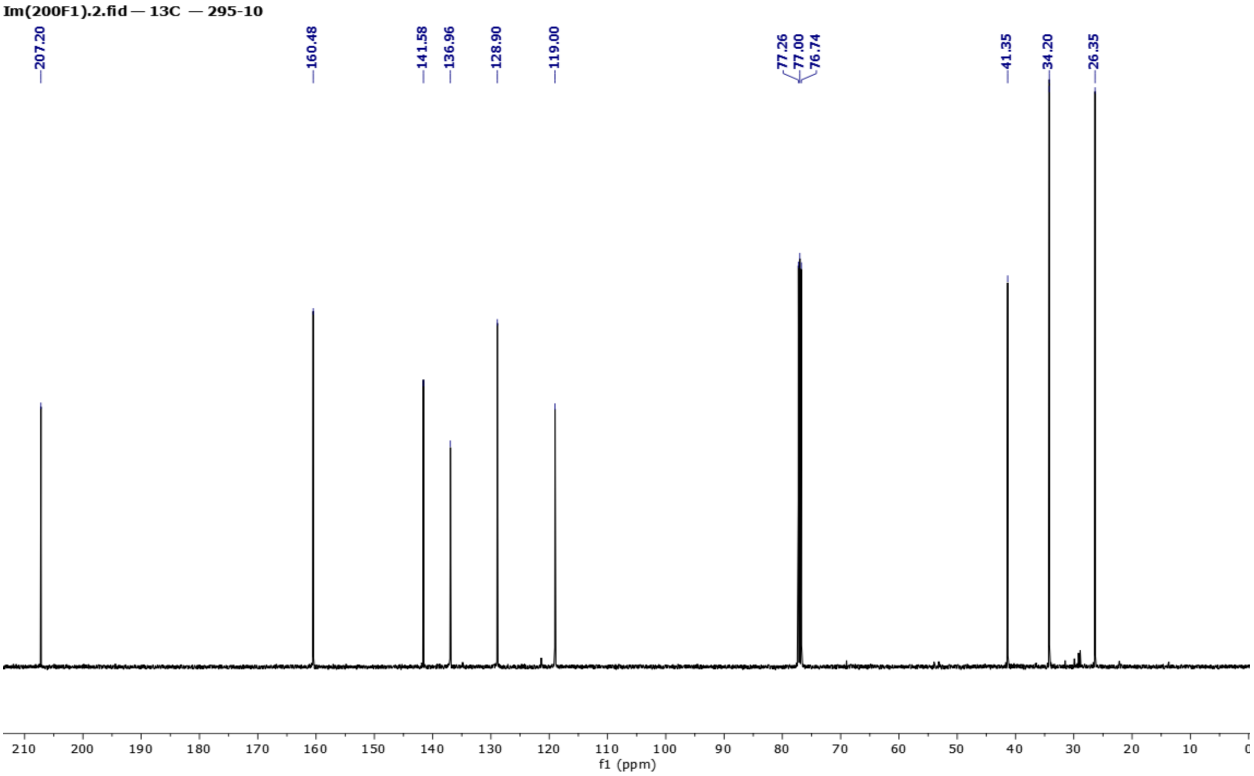

Figure S4. ^{13}C NMR (125 MHz, $CDCl_3$) spectra of **6b**

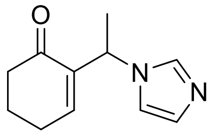

6c

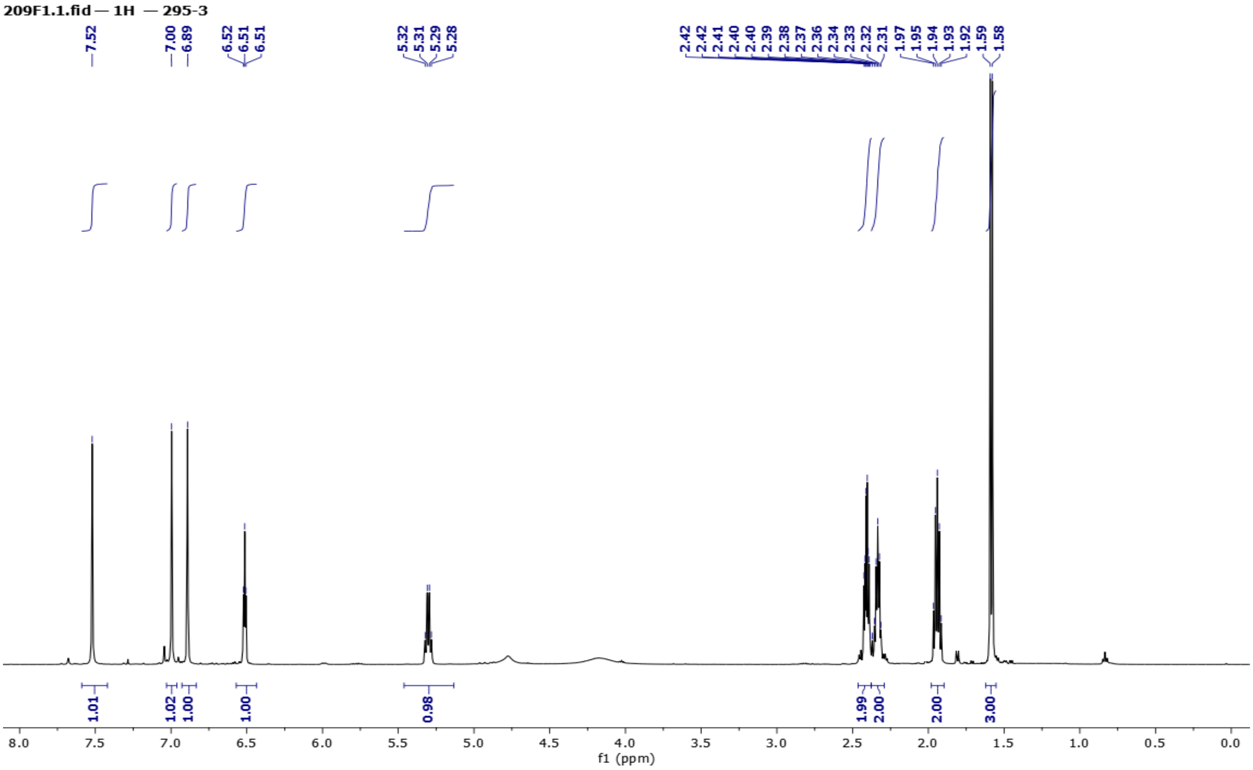

Figure S5. ^{1}H NMR (500 MHz, $CDCl_3$) spectra of **6c**

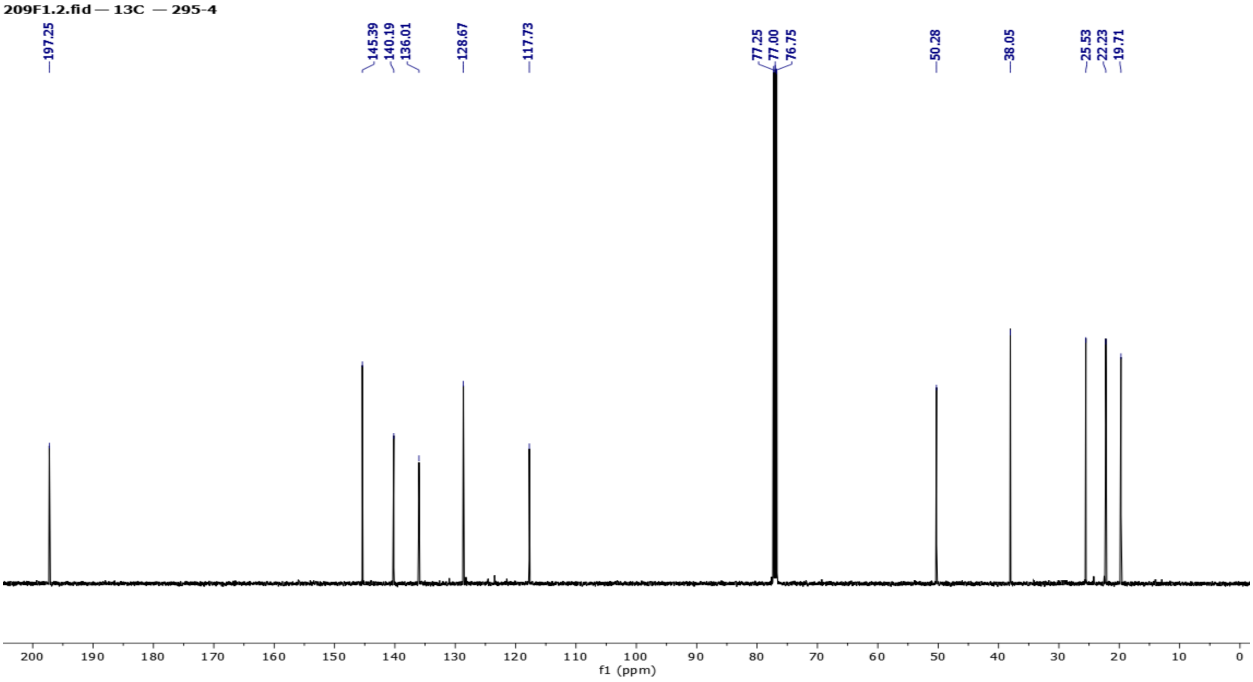

Figure S6. ^{13}C NMR (125 MHz, $CDCl_3$) spectra of **6c**

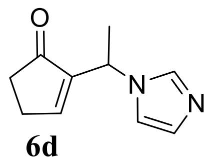

6d

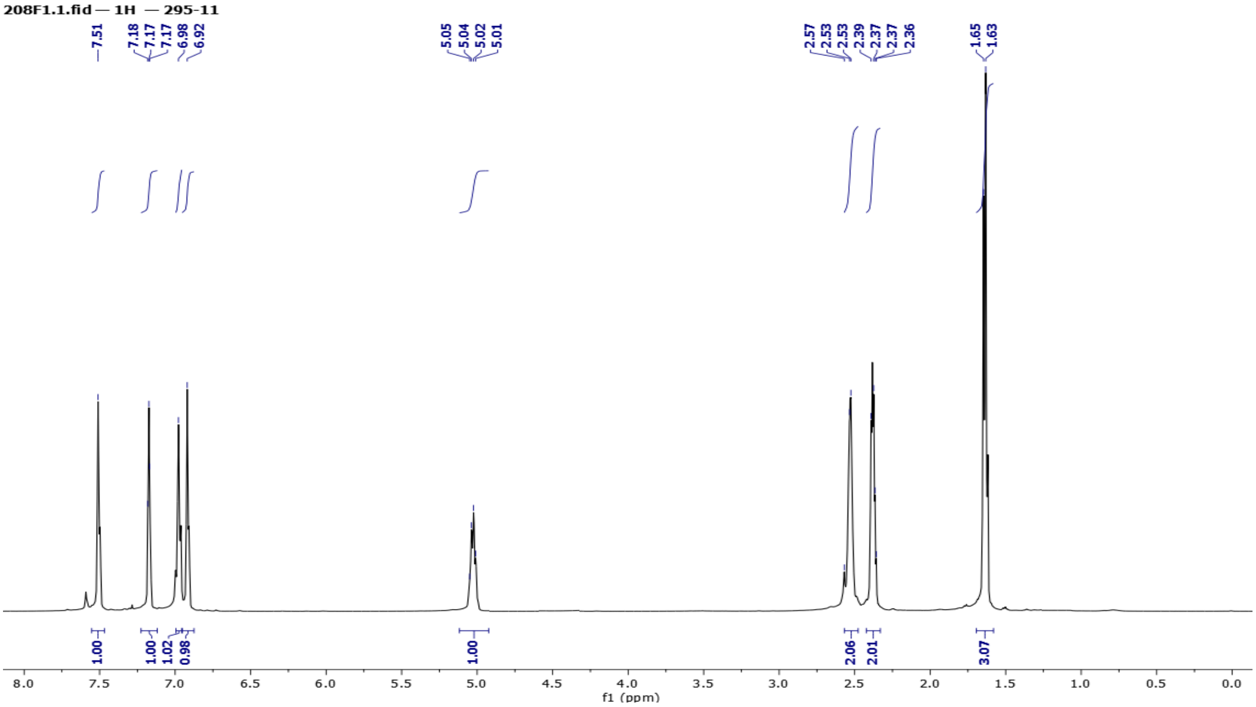

Figure S7. ^{1}H NMR (500 MHz, $CDCl_3$) spectra of **6d**

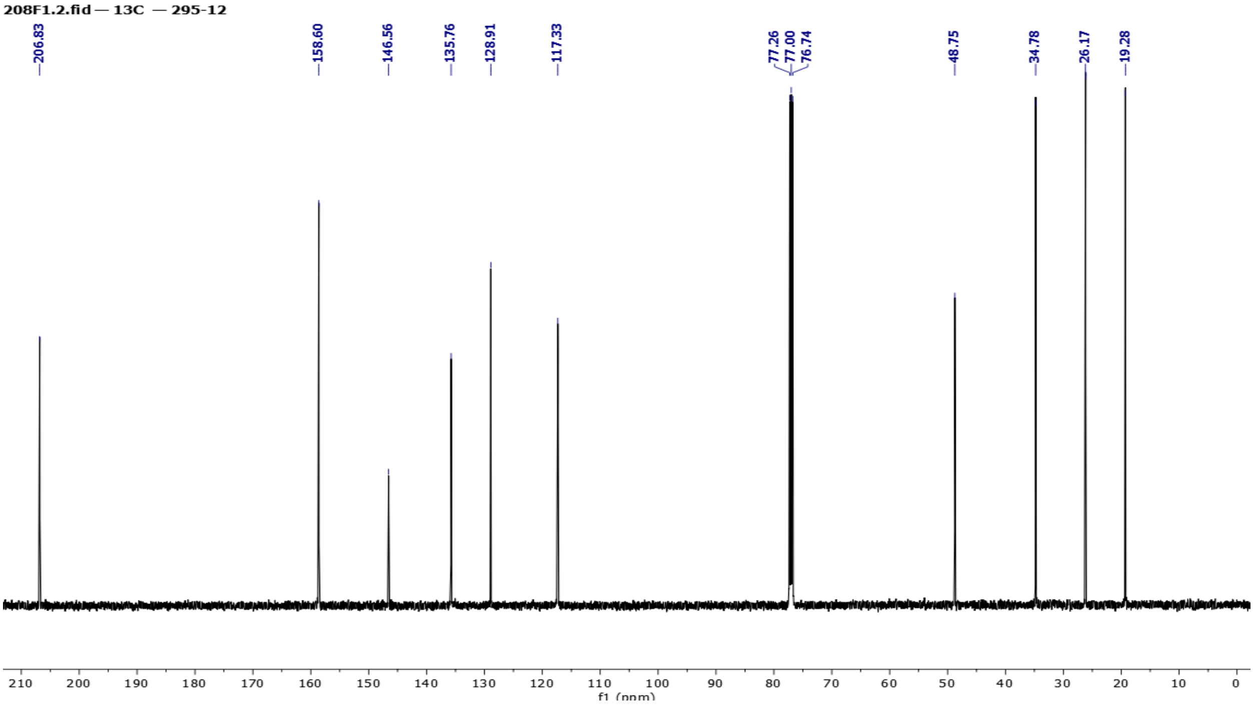

Figure S8. ^{13}C NMR (125 MHz, $CDCl_3$) spectra of **6d**

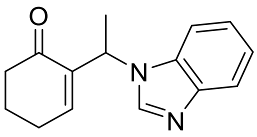

7a

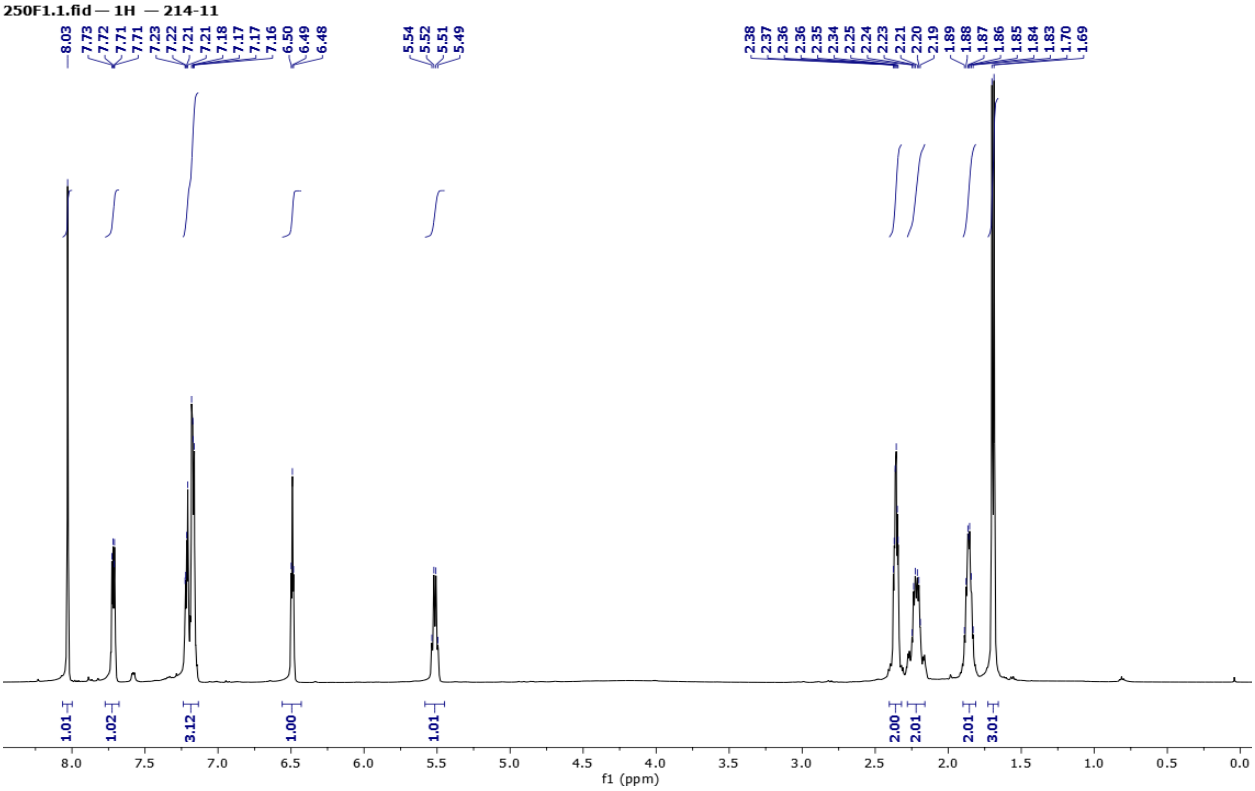

Figure S9. ${}^{1}H$ NMR (500 MHz, $CDCl_3$) spectra of **7a**

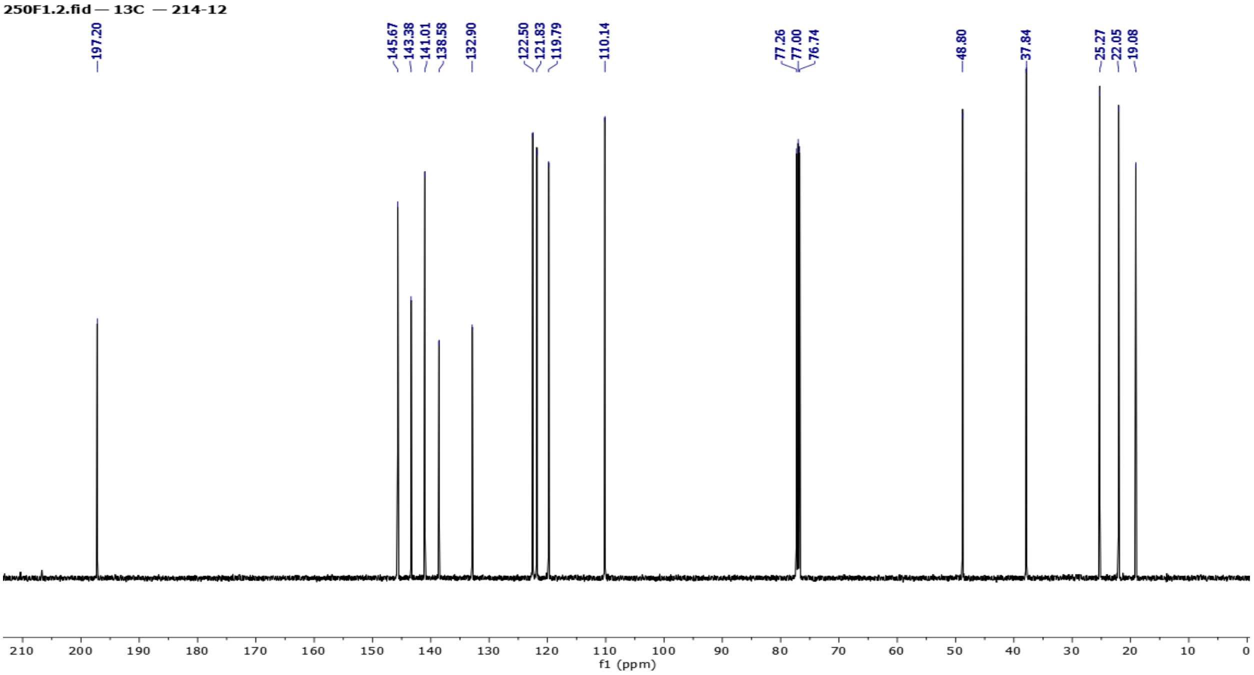

Figure S10. ${}^{13}C$ NMR (125 MHz, $CDCl_3$) spectra of **7a**

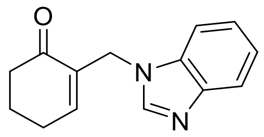

7b

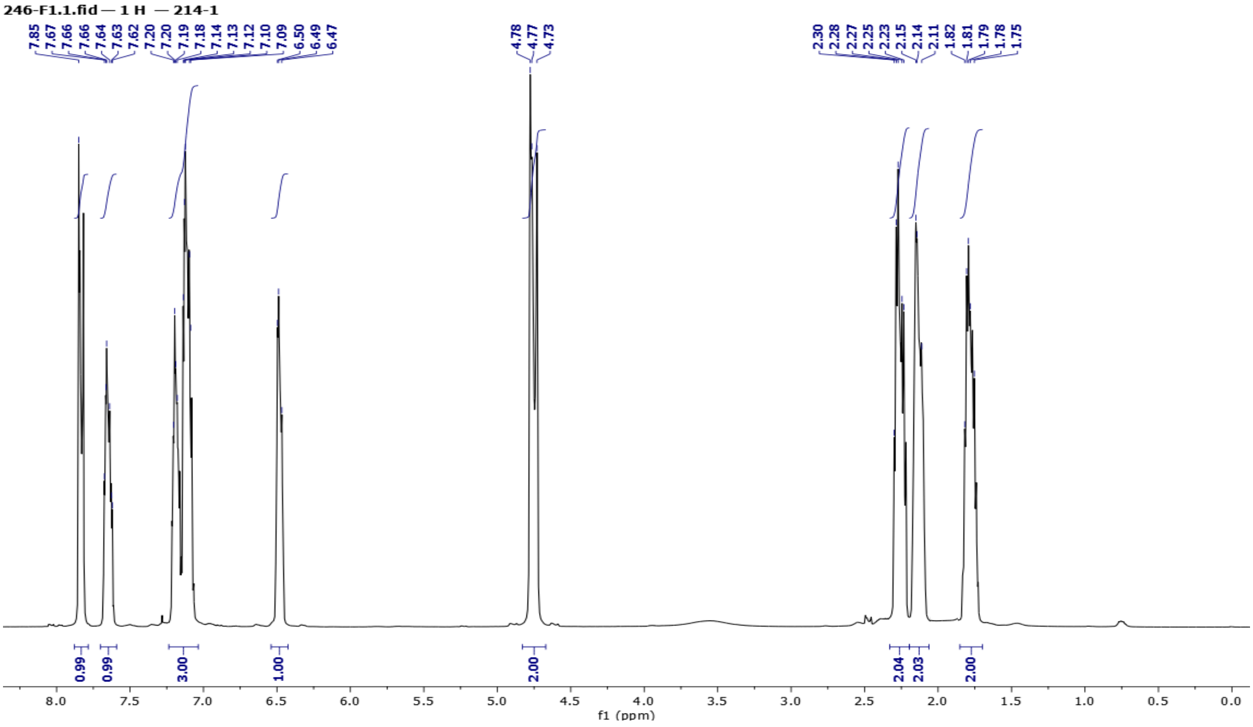

Figure S11. ^{1}H NMR (500 MHz, $CDCl_3$) spectra of **7b**

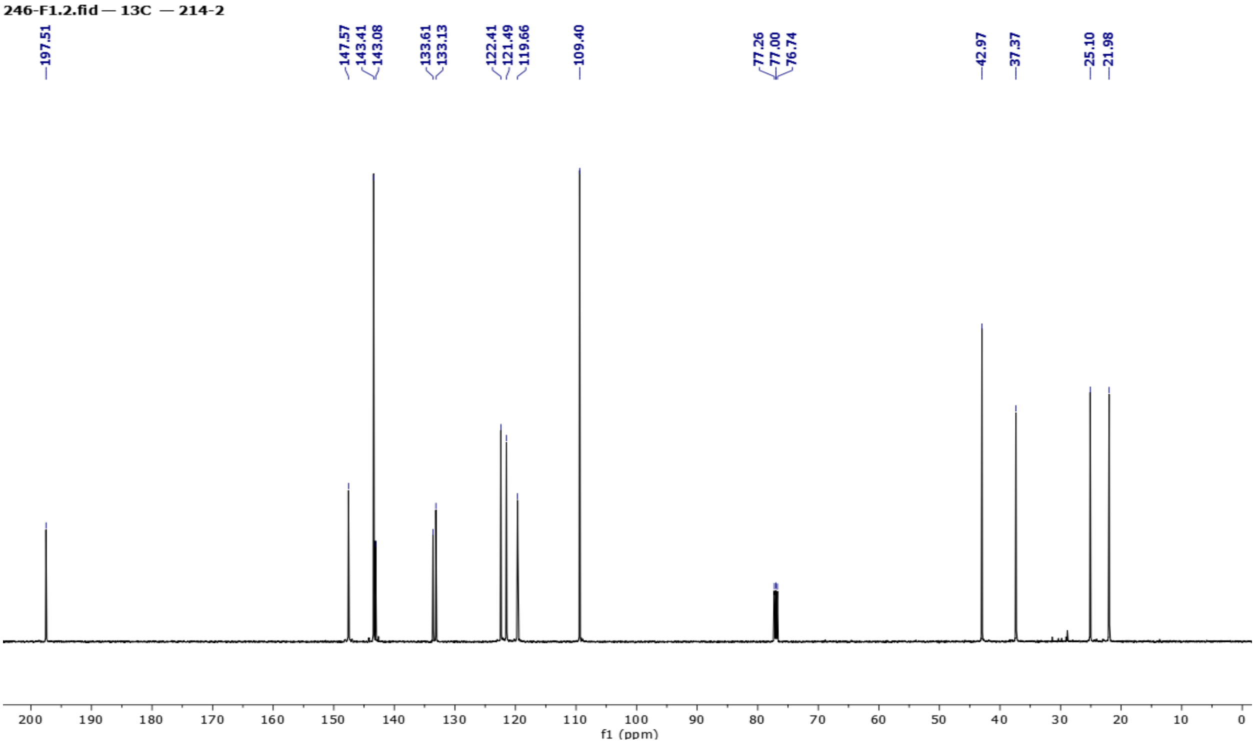

Figure S12. ^{13}C NMR (125 MHz, $CDCl_3$) spectra of **7b**

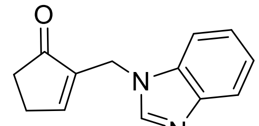

7c

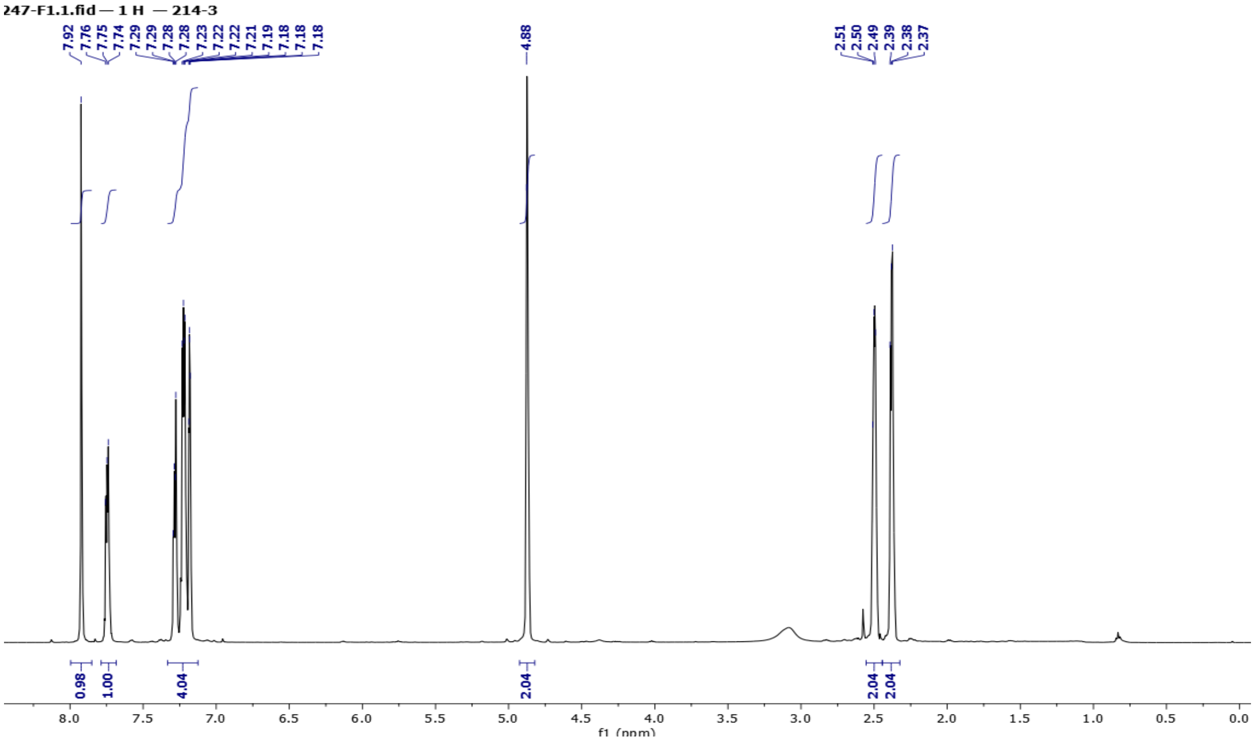

Figure S13. ^{1}H NMR (500 MHz, $CDCl_3$) spectra of **7c**

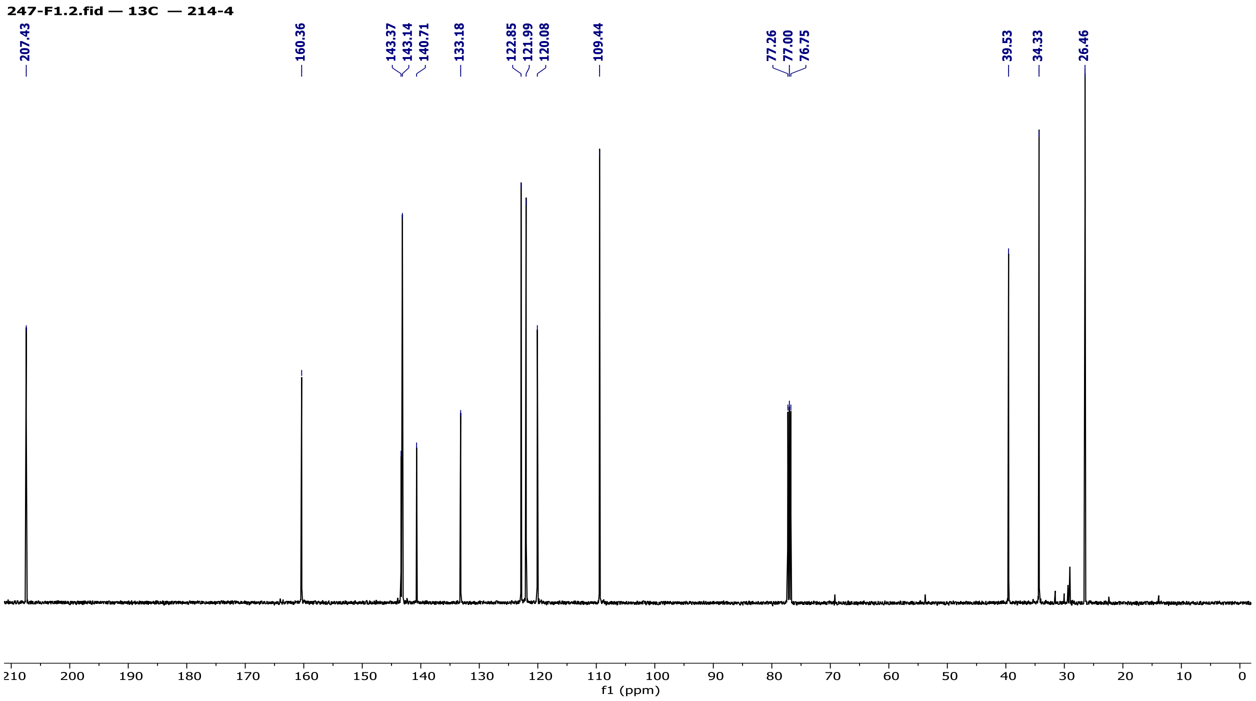

Figure S14. ^{13}C NMR (125 MHz, $CDCl_3$) spectra of **7c**

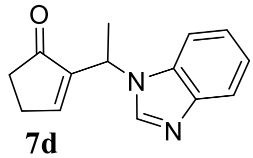

7d

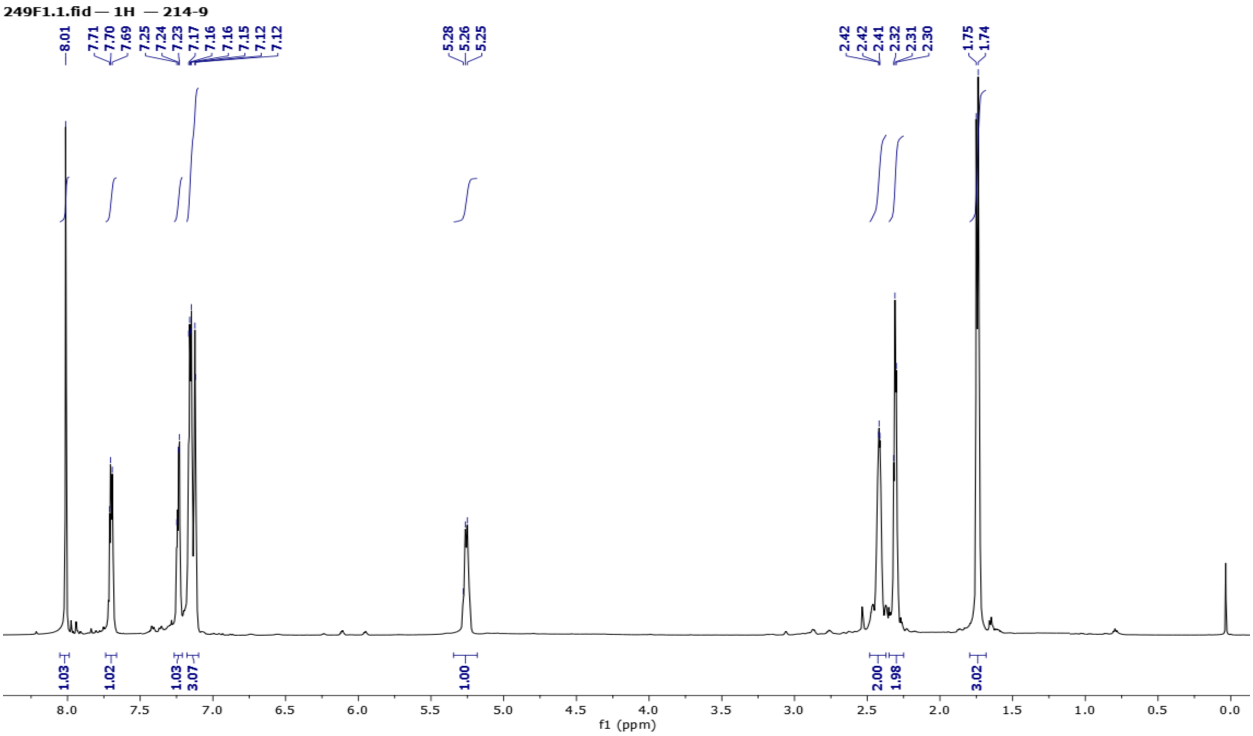

Figure S15. ^{1}H NMR (500 MHz, $CDCl_3$) spectra of **7d**

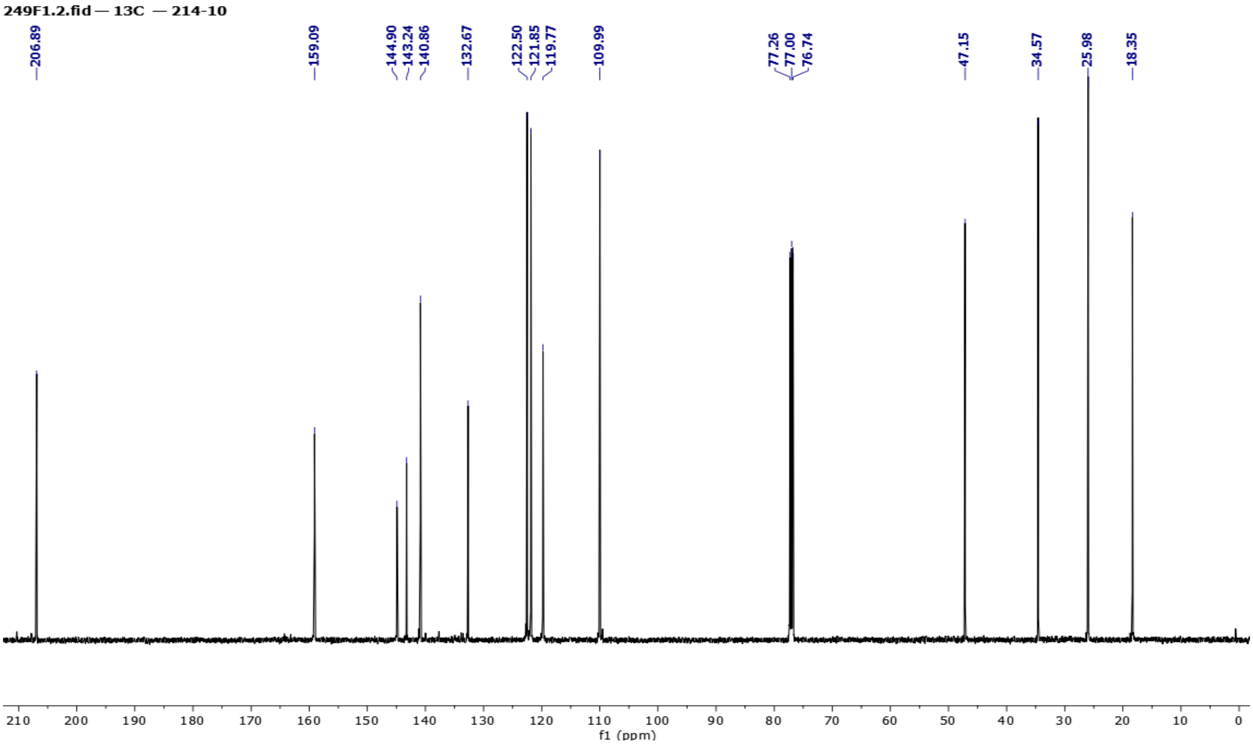

Figure S16. ^{13}C NMR (125 MHz, $CDCl_3$) spectra of **7d**

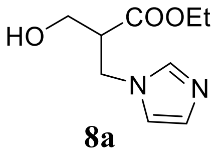

8a

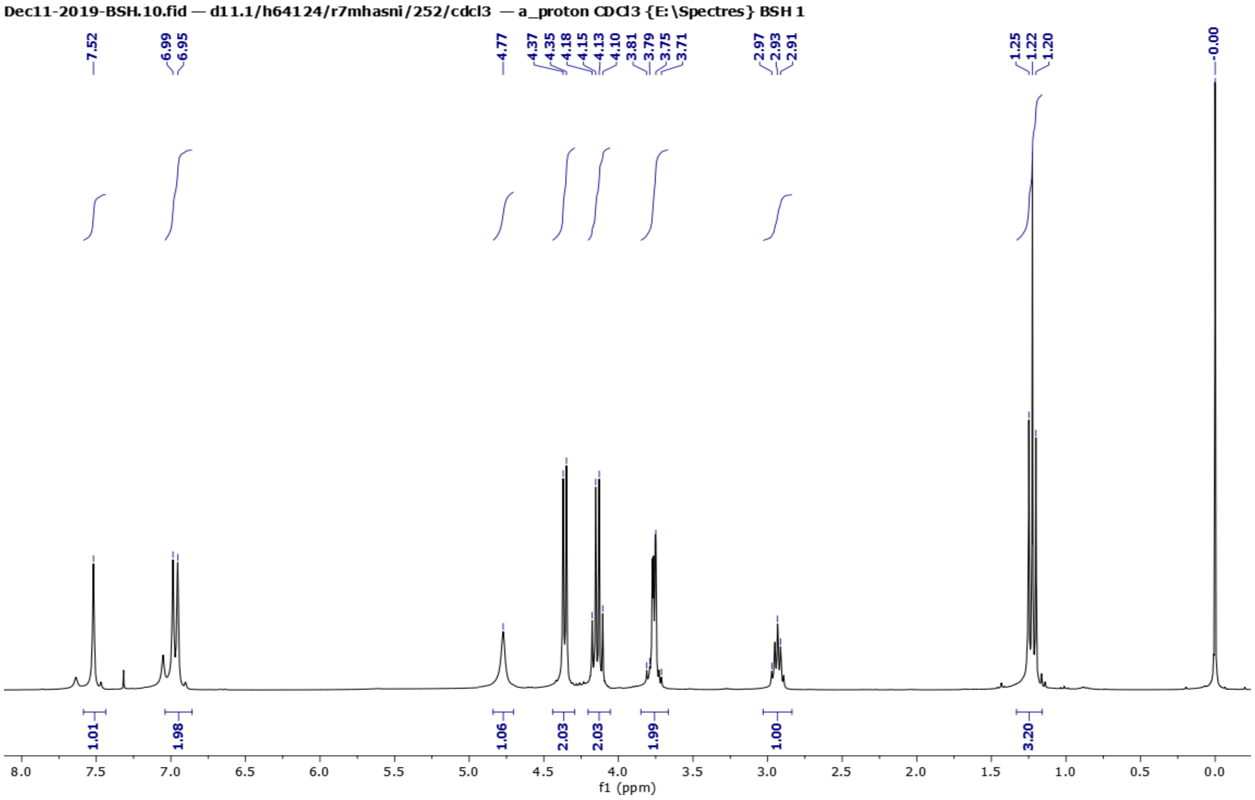

Figure S17. ^{1}H NMR (300 MHz, $CDCl_3$) spectra of **8a**

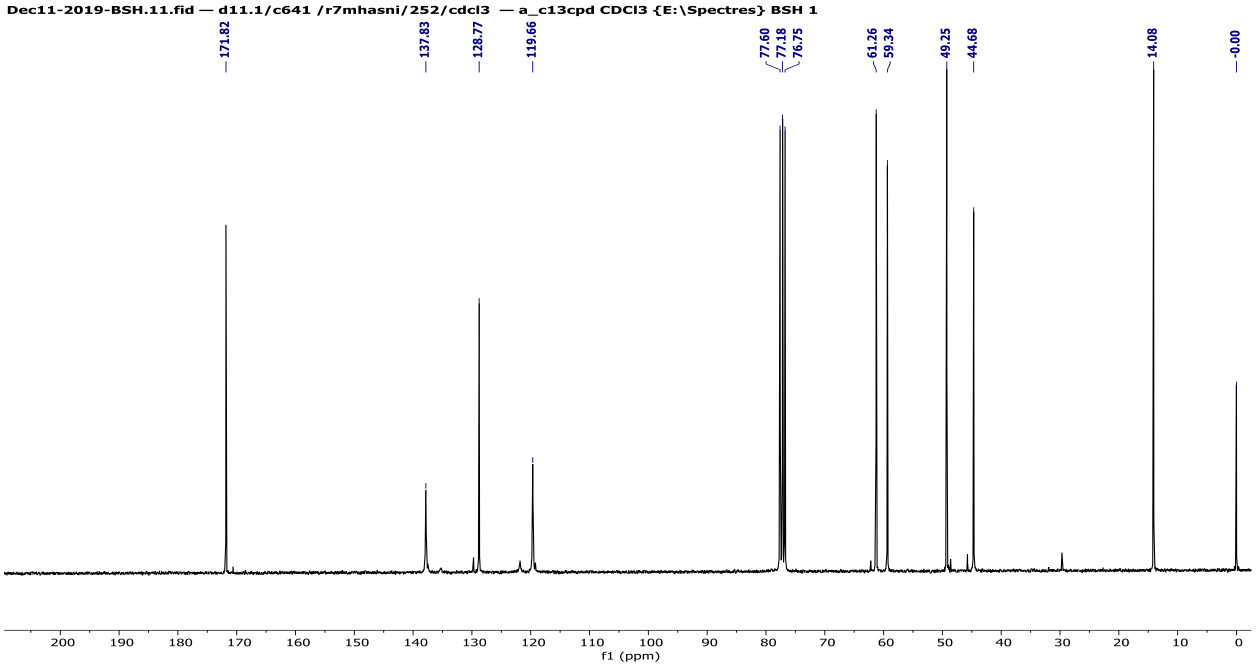

Figure S18. ^{13}C NMR (75 MHz, $CDCl_3$) spectra of **8a**

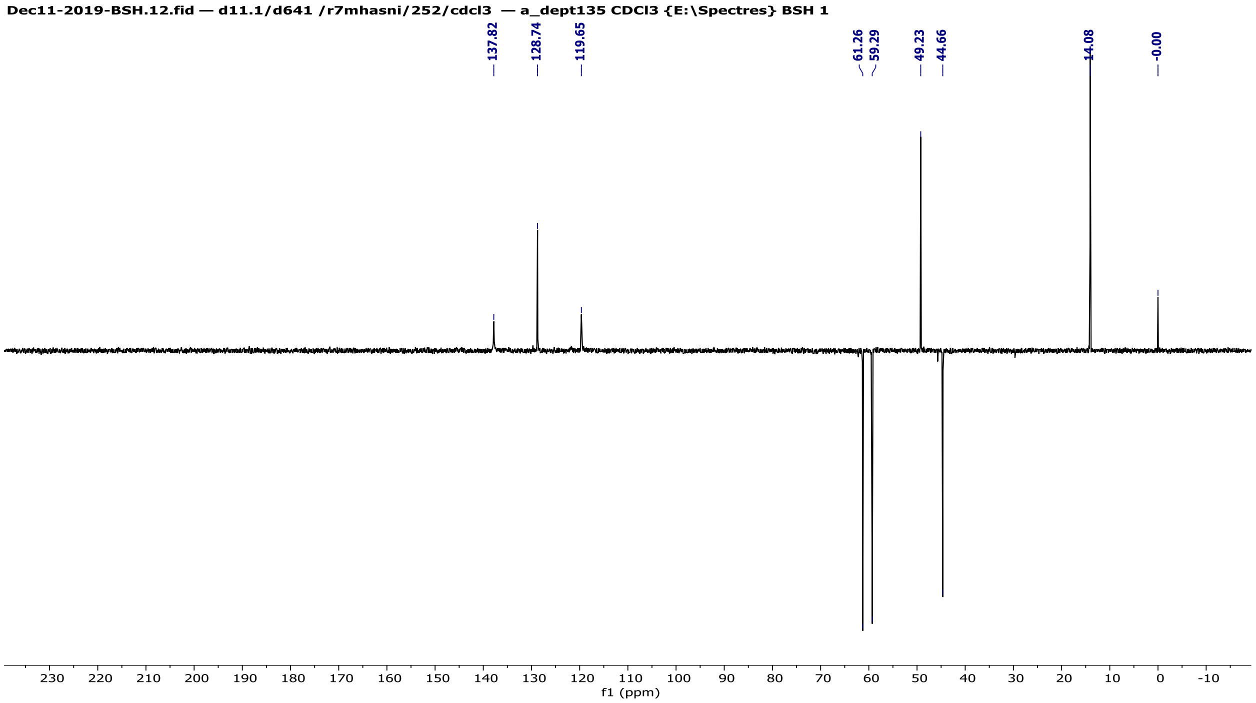

Figure S19. dept 135 NMR (75 MHz, $CDCl_3$) spectra of **8a**

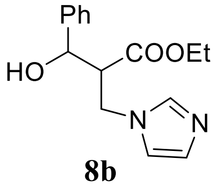

8b

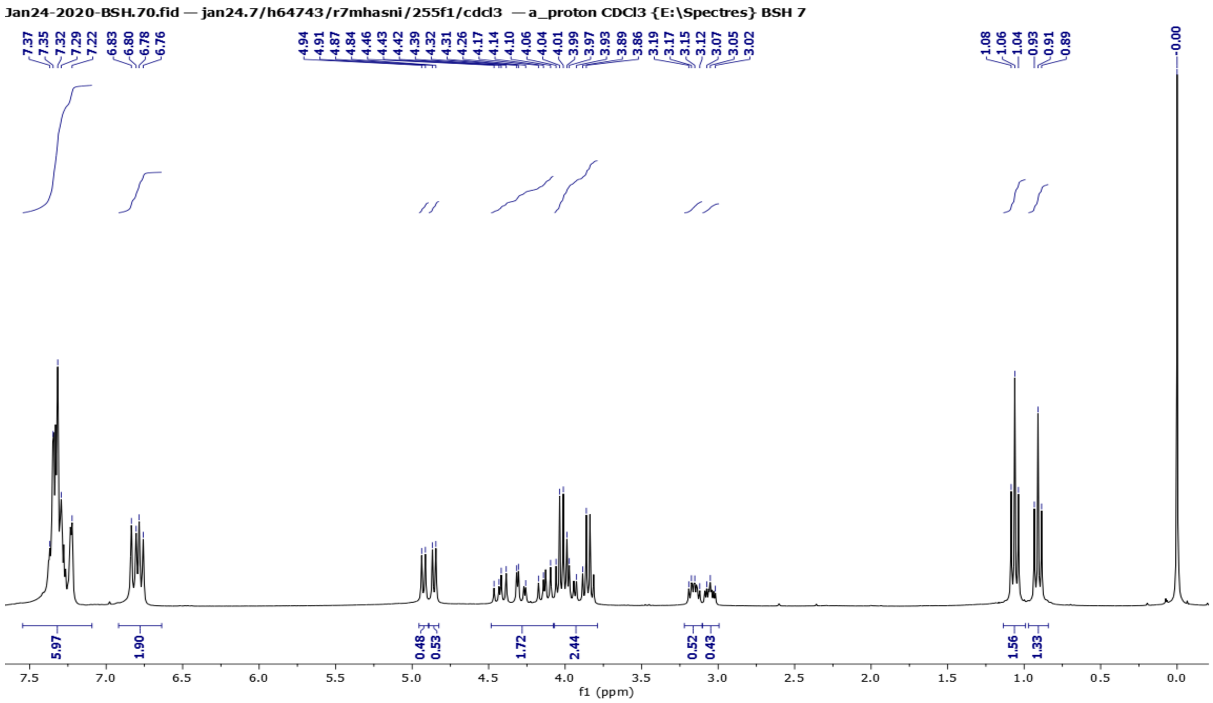

Figure S20. ^{1}H NMR (300 MHz, $CDCl_3$) spectra of **8b**

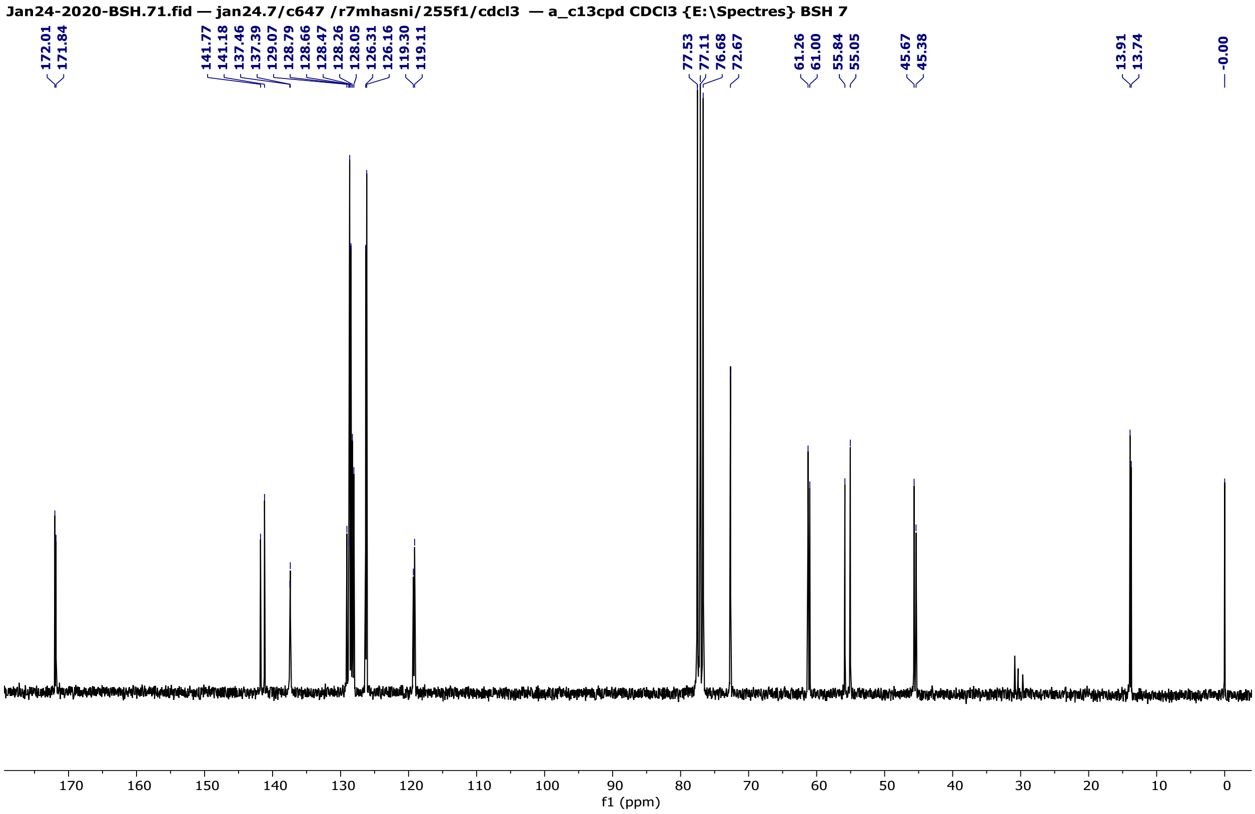

Figure S21. ^{13}C NMR (75 MHz, $CDCl_3$) spectra of **8b**

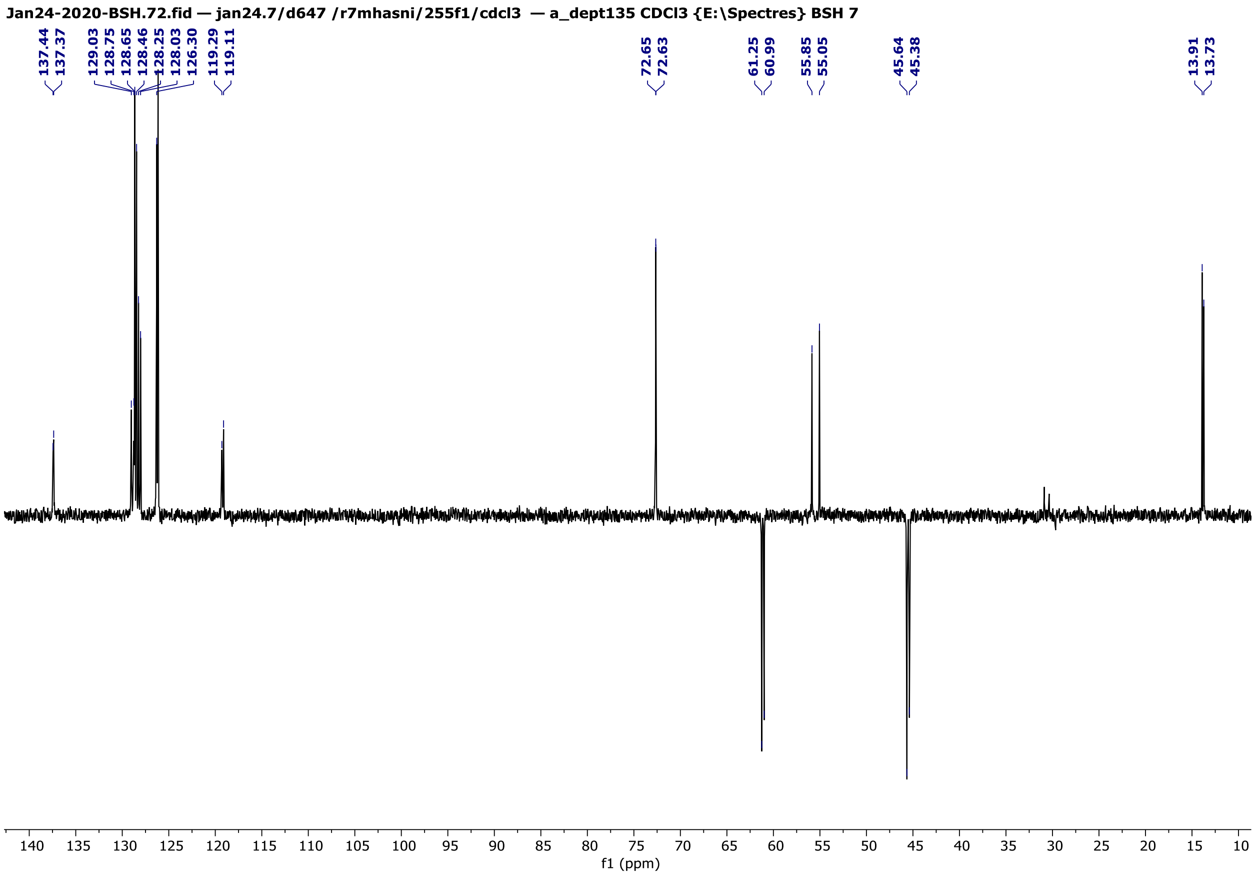

Figure S22. dept 135 NMR (75 MHz, $CDCl_3$) spectra of **8b**

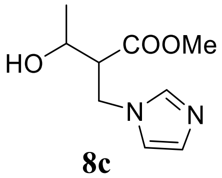

8c

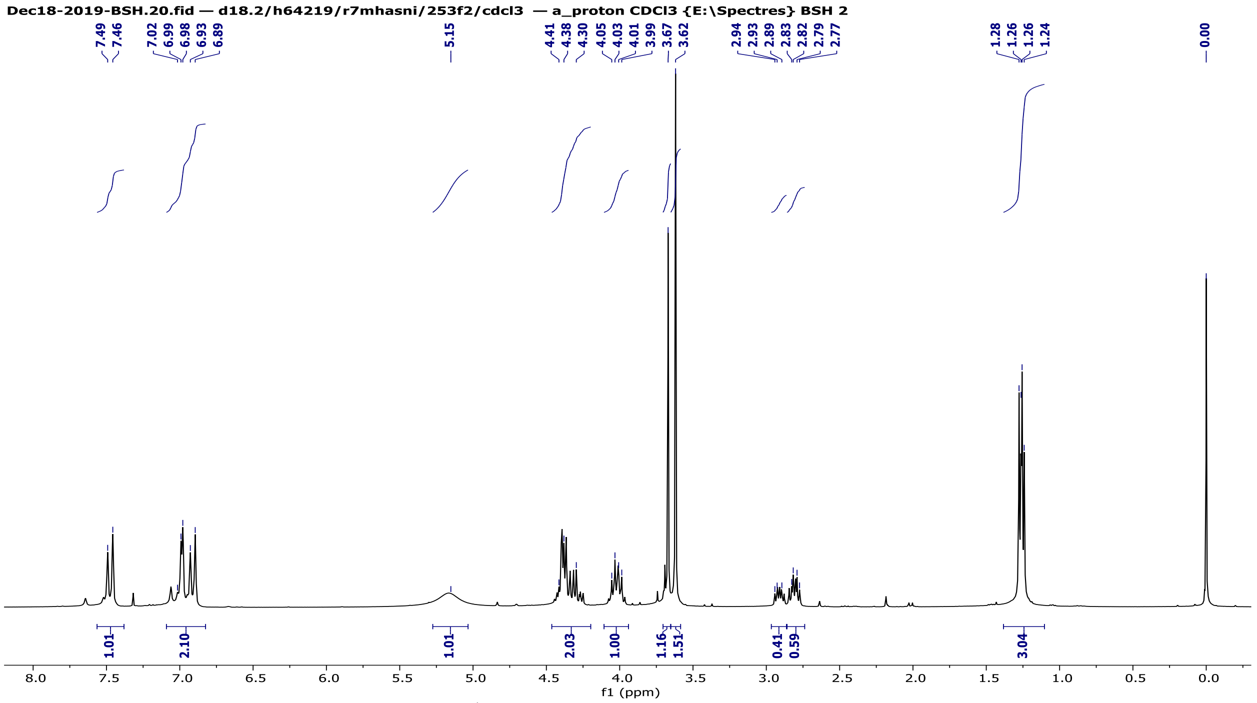

Figure S23. ^{1}H NMR (300 MHz, $CDCl_3$) spectra of **8c**

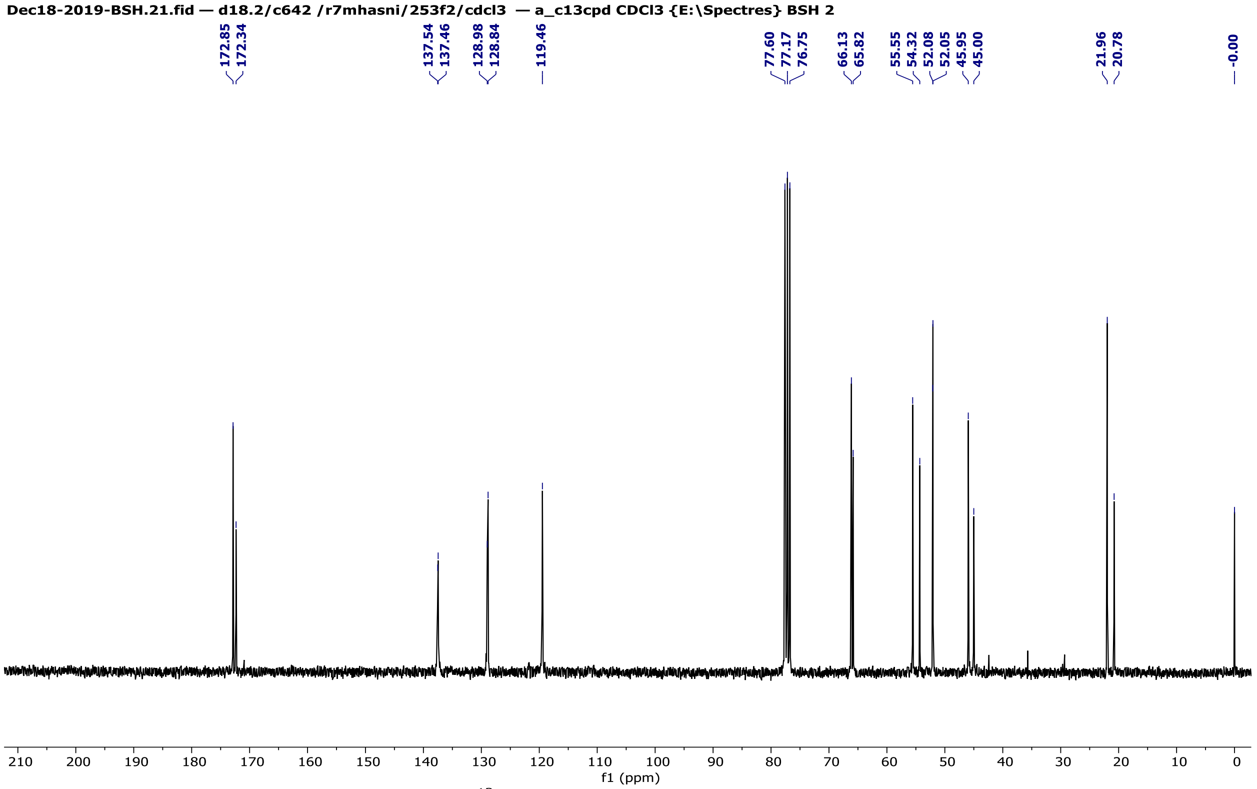

Figure S24. ^{13}C NMR (75 MHz, $CDCl_3$) spectra of **8c**

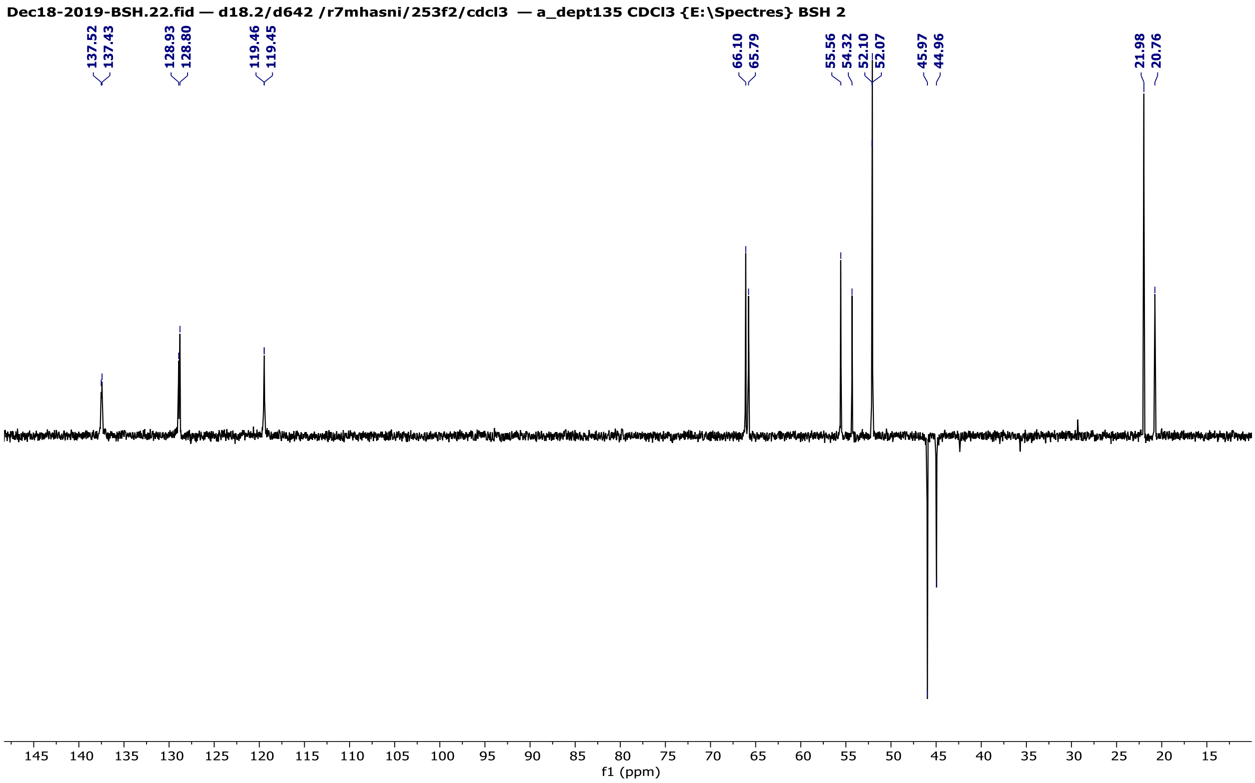

Figure S25. dept 135 NMR (75 MHz, $CDCl_3$) spectra of **8c**

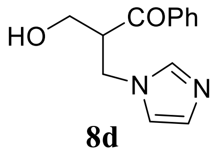

8d

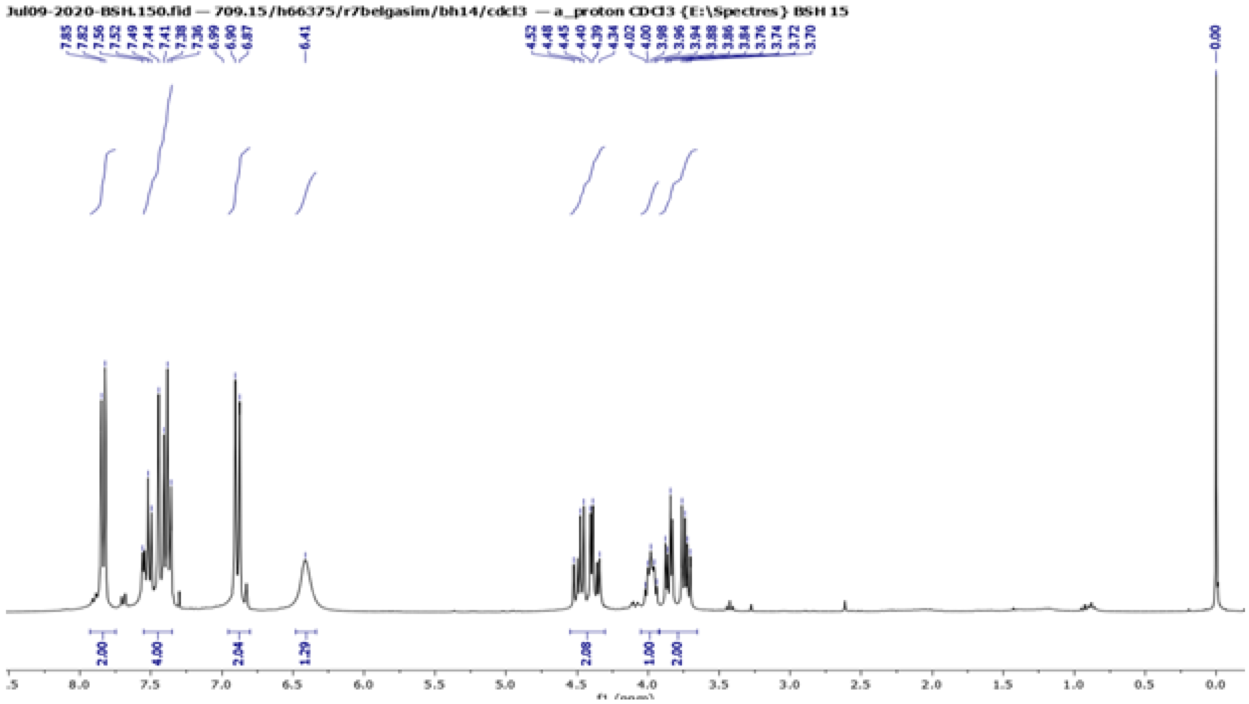

Figure S26. ^{1}H NMR (300 MHz, $CDCl_3$) spectra of **8d**

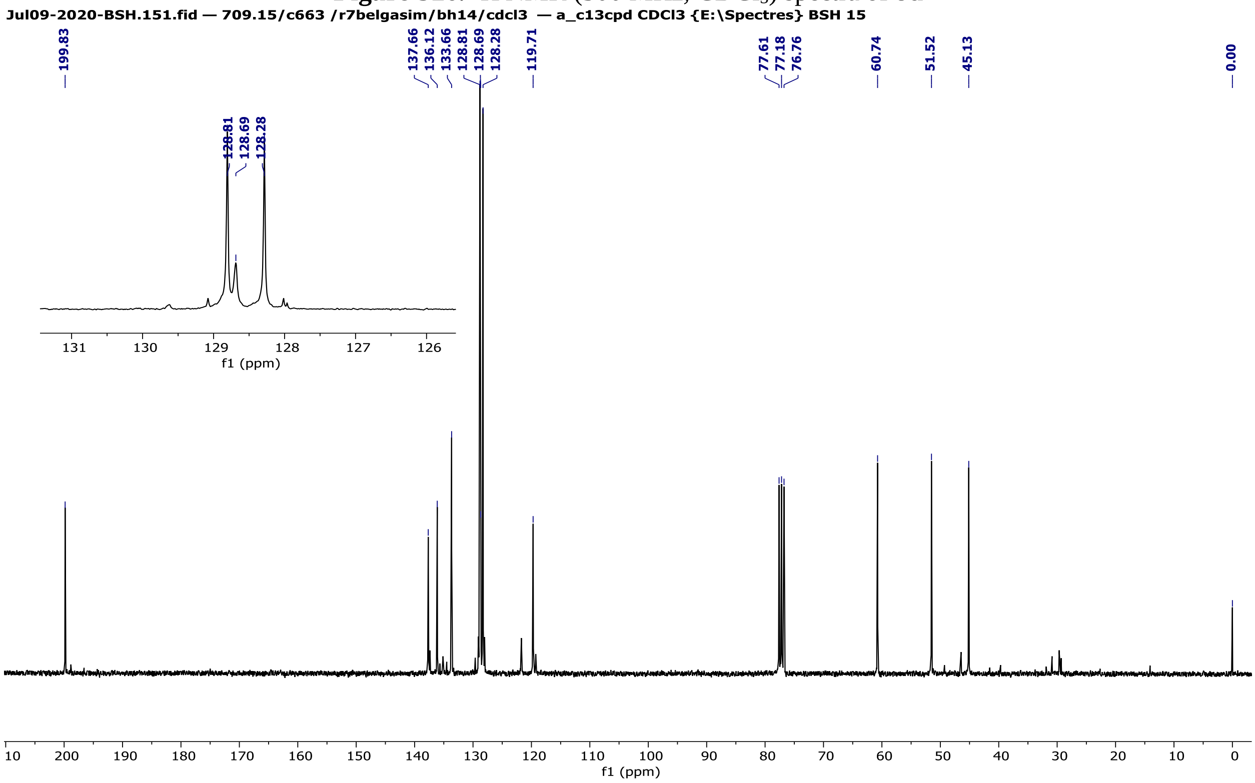

Figure S27. ^{13}C NMR (75 MHz, $CDCl_3$) spectra of **8d**

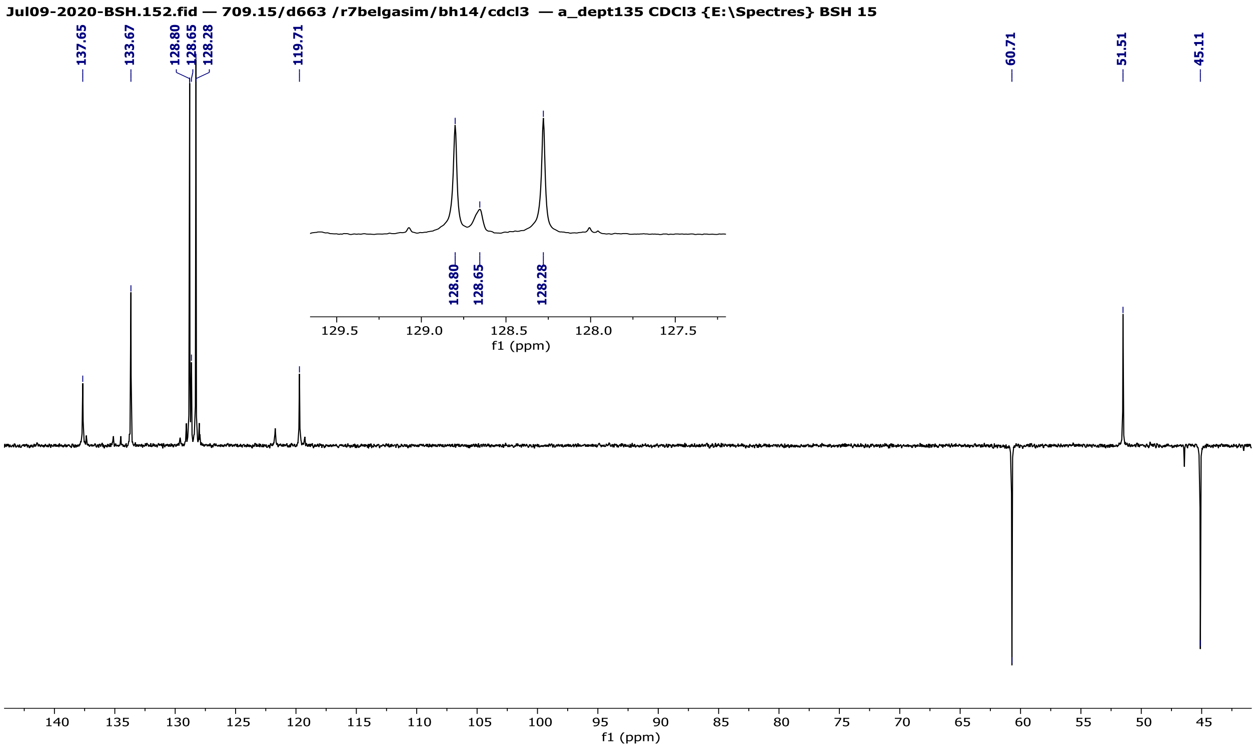

Figure S28. dept 135 NMR (75 MHz, $CDCl_3$) spectra of **8d**

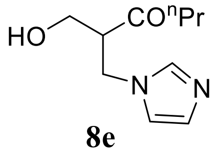

8e

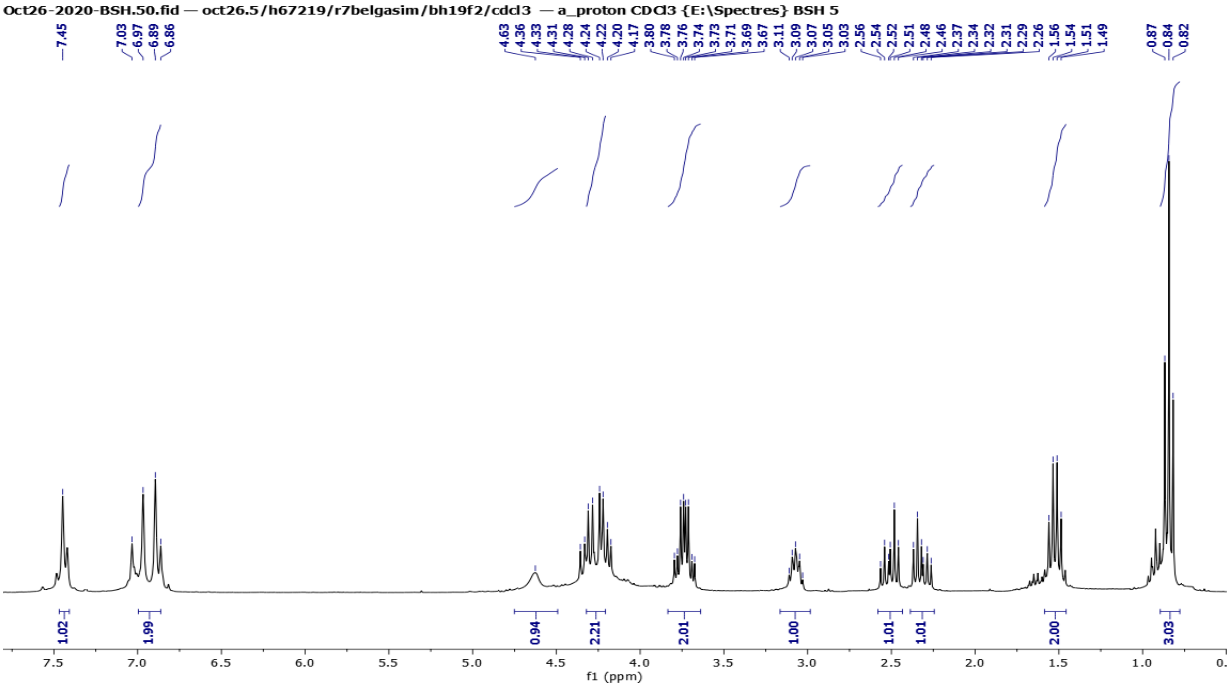

Figure S29. ^{1}H NMR (300 MHz, $CDCl_3$) spectra of **8e**

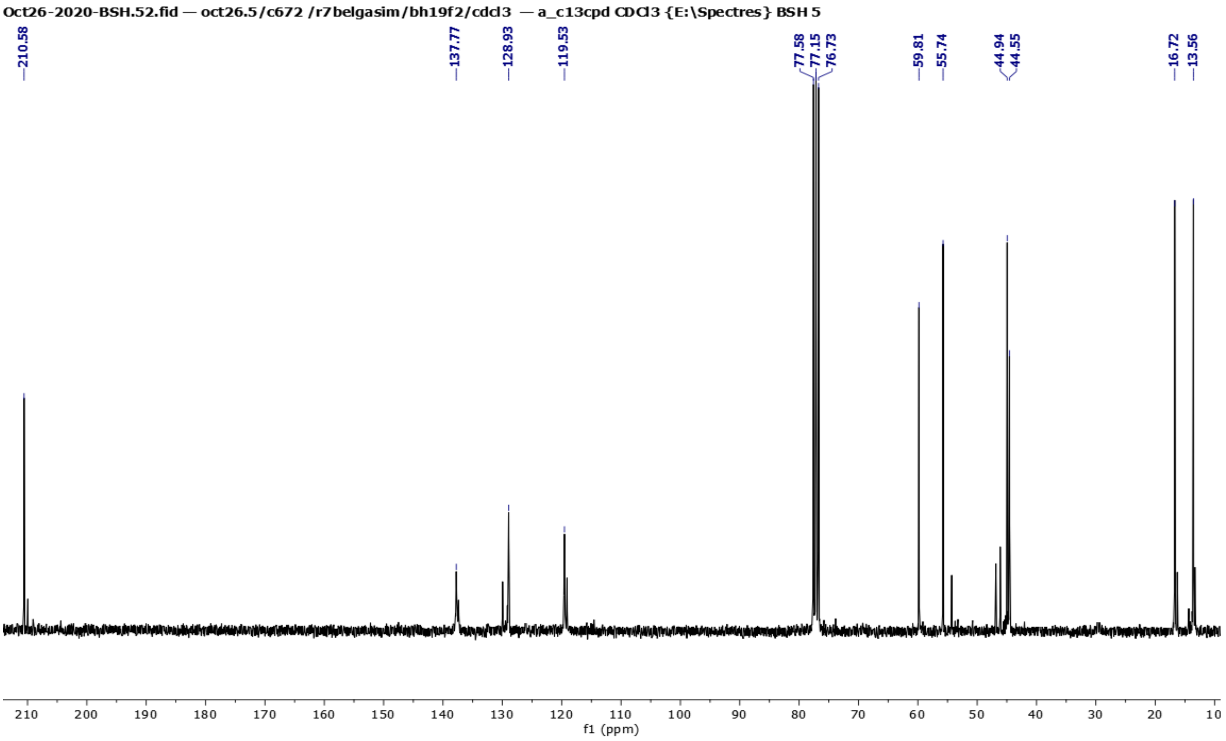

Figure S30. ^{13}C NMR (75 MHz, $CDCl_3$) spectra of **8e**

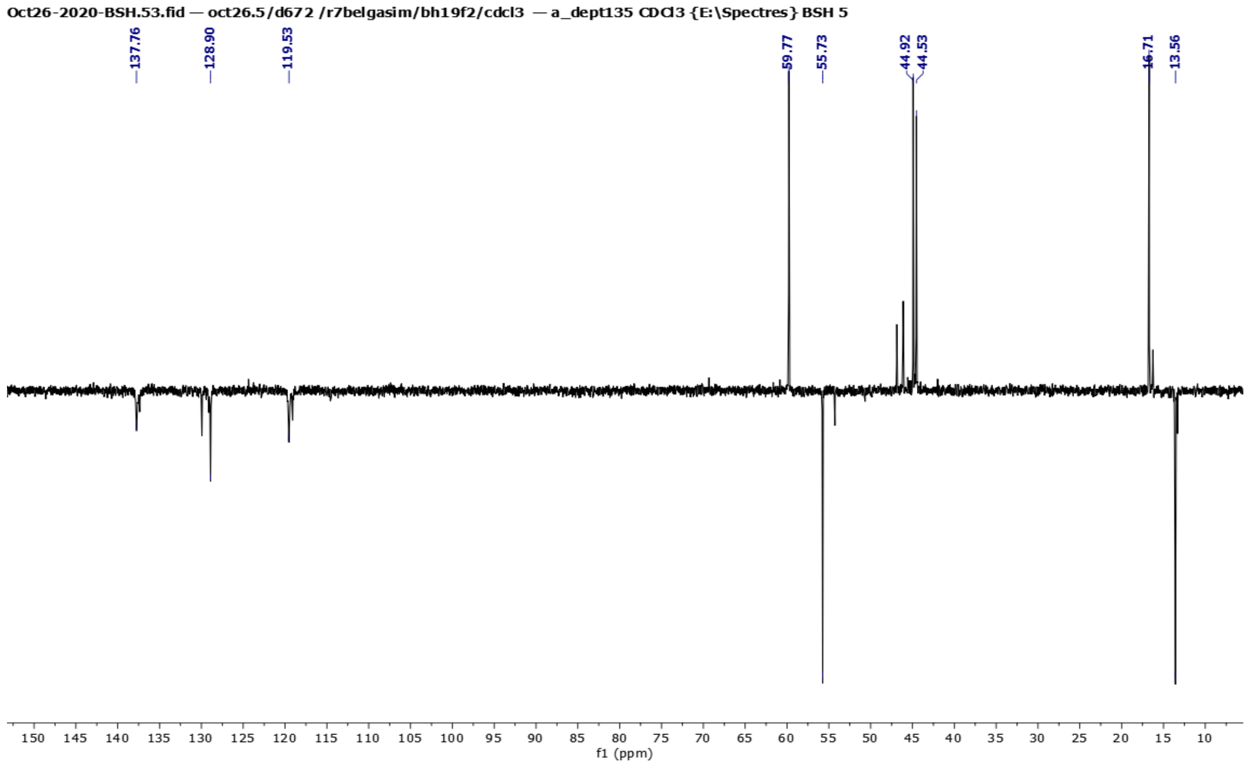

Figure S31. dept 135 NMR (75 MHz, $CDCl_3$) spectra of **8e**

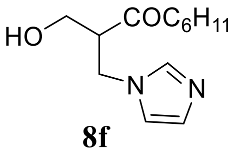

8f

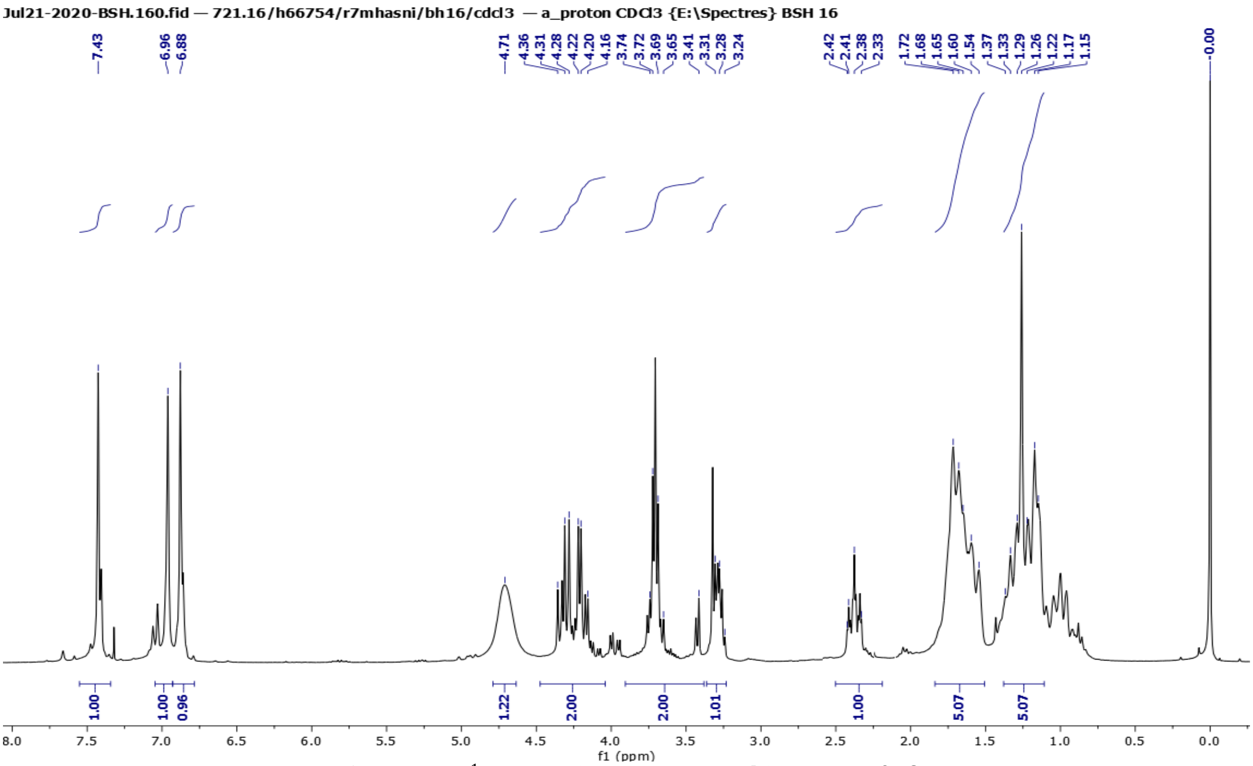

Figure S32. ^{1}H NMR (300 MHz, $CDCl_3$) spectra of **8f**

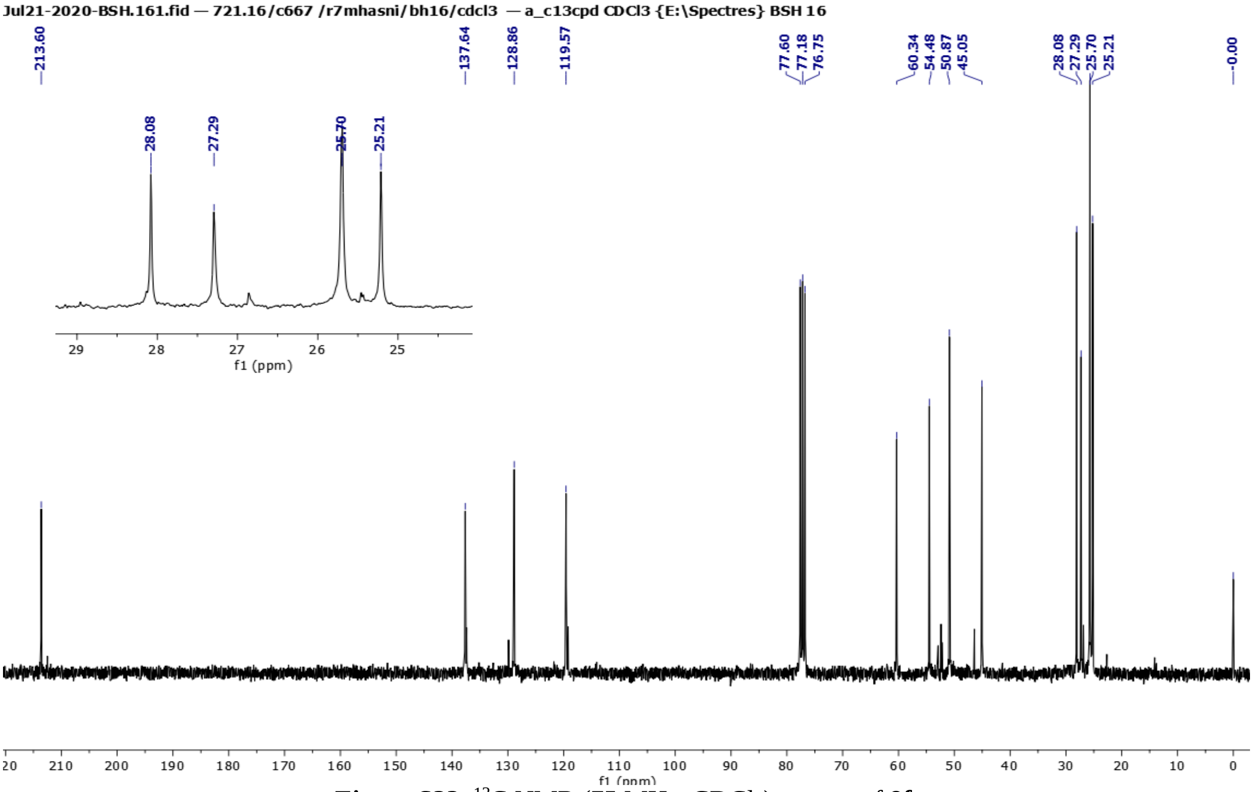

Figure S33. ^{13}C NMR (75 MHz, $CDCl_3$) spectra of **8f**

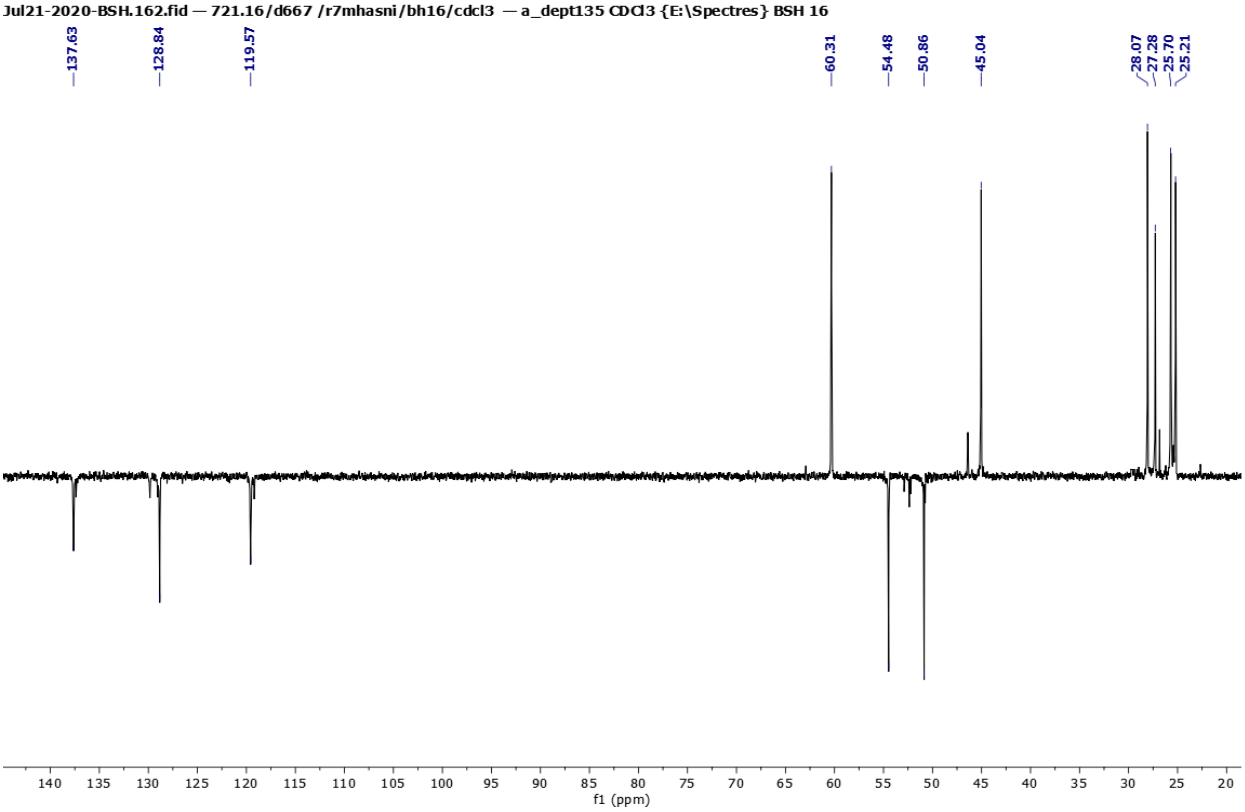

Figure S34. dept 135 NMR (75 MHz, $CDCl_3$) spectra of **8f**

ESI-HRMS spectra for compounds 6a–d, 7a–d, 8a–f

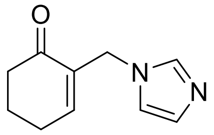

6a

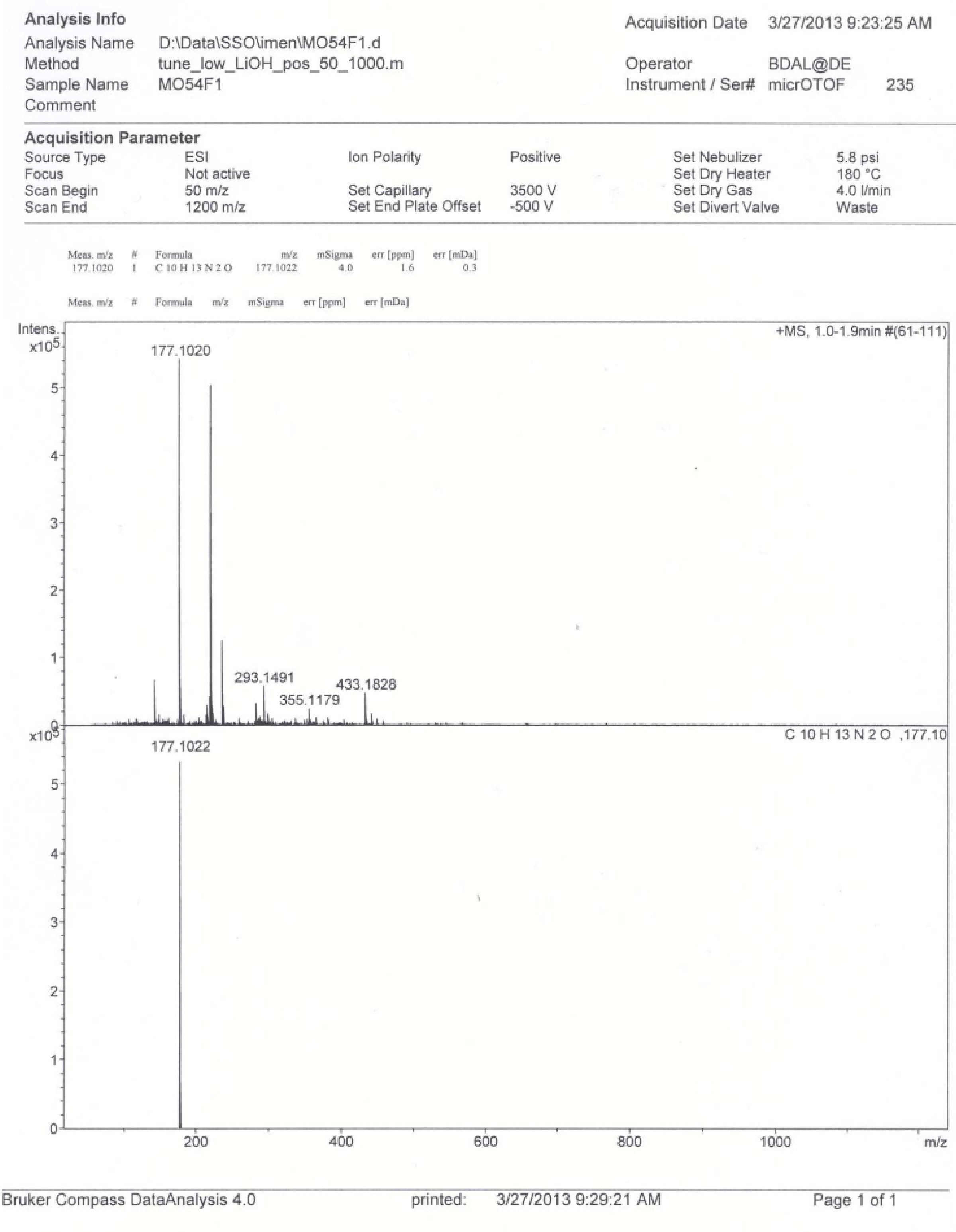

Figure S35. ESI-HRMS spectra of **6a**

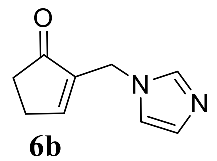

6b

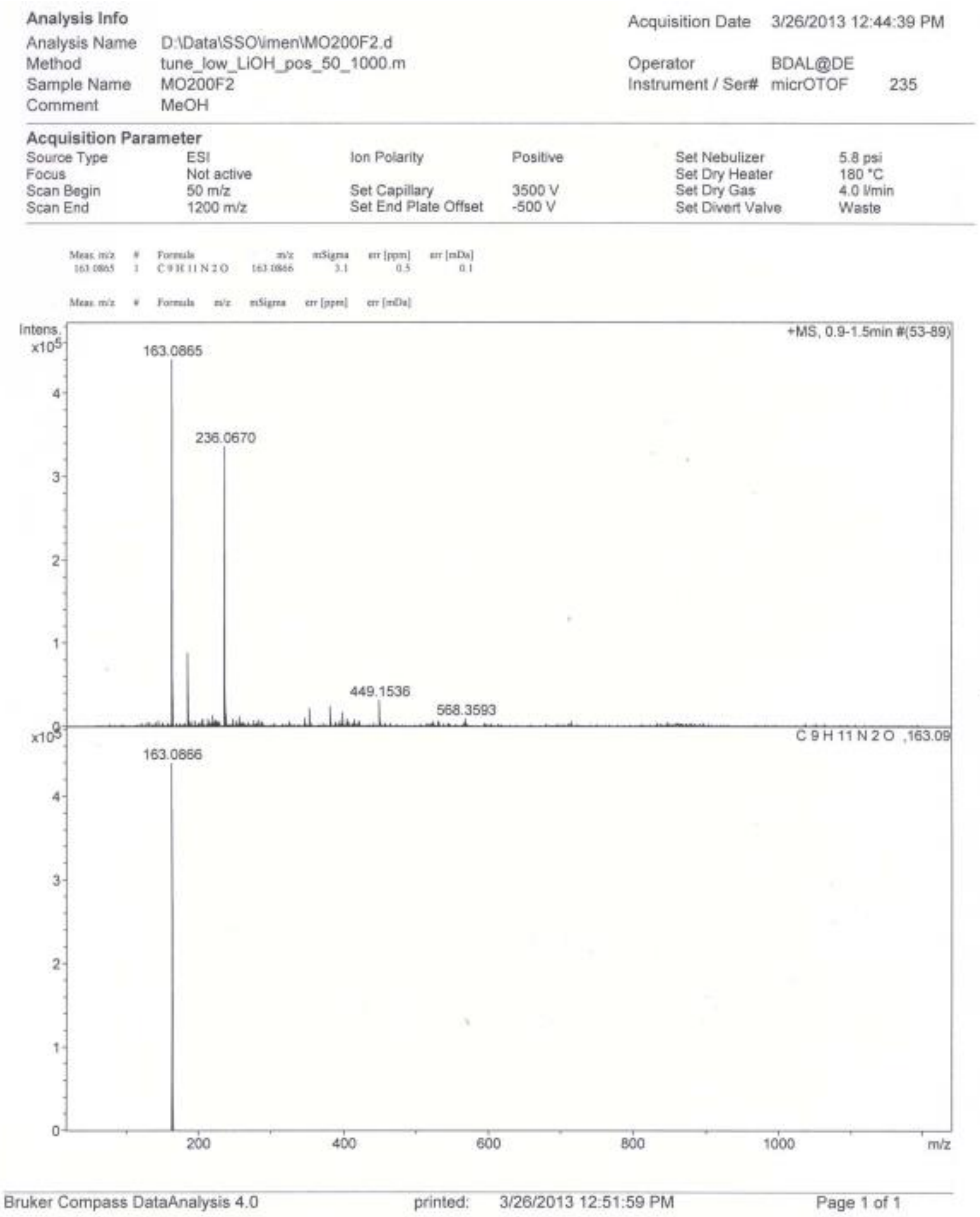

Figure S36. ESI-HRMS spectra of **6b**

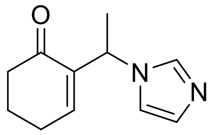

6c

Elemental Composition Report

Page 1

Single Mass Analysis

Tolerance = 3.0 PPM / DBE: min = -5.0, max = 80.0
Element prediction: Off
Number of isotope peaks used for i-FIT = 3

Monoisotopic Mass, Even Electron Ions
226 formula(e) evaluated with 1 results within limits (up to 50 closest results for each mass)
Elements Used:
C: 0-80 H: 0-100 N: 0-10 O: 0-10

Cone voltage =30V Xevo G2 QTof #YCA210 07-Jul-2022 09:20:01
205F1 75 (0.471) AM2 (Ar,20000.0,0.00,0.00); Cm (67:75-62:65x2.000) 1: TOF MS ES+ 3.68e+006

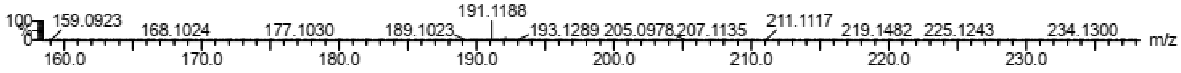

Minimum:				-5.0				
Maximum:		3.0	3.0	80.0				
Mass	Calc. Mass	mDa	PPM	DBE	i-FIT	Norm	Conf(%)	Formula
191.1188	191.1184	0.4	2.1	5.5	700.8	n/a	n/a	C11 H15 N2 O

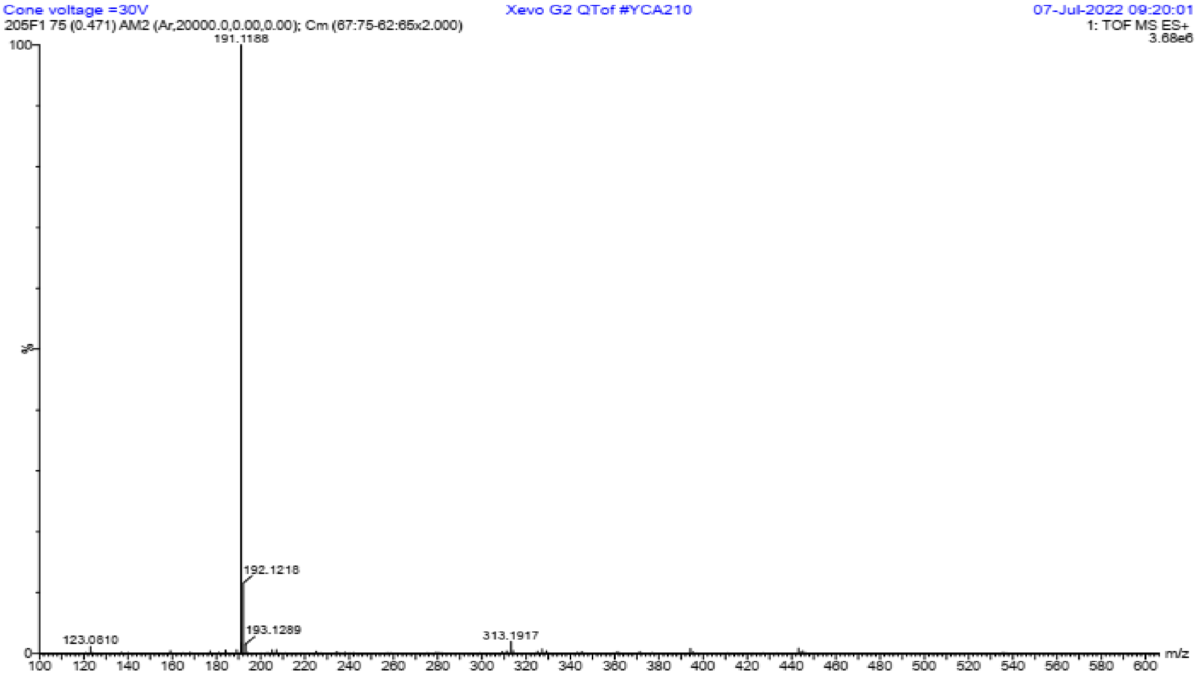

Figure S37. ESI-HRMS spectra of **6c**

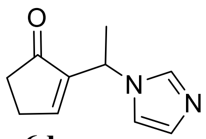

6d

Elemental Composition Report **Page 1**

Single Mass Analysis
Tolerance = 3.0 PPM / DBE: min = -5.0, max = 80.0
Element prediction: Off
Number of isotope peaks used for i-FIT = 3

Monoisotopic Mass, Even Electron Ions
196 formula(e) evaluated with 1 results within limits (up to 50 closest results for each mass)
Elements Used:
C: 0-80 H: 0-100 N: 0-10 O: 0-10

Cone voltage =30V Xevo G2 QTof #YCA210 05-Jul-2022 15:33:15
208F1 74 (0.465) AM2 (Ar,20000.0,0.00,0.00); Cm (70:74-54:64x2.000) 1: TOF MS ES+
1.57e+006

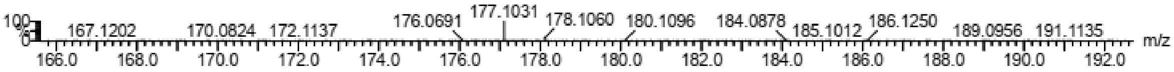

Minimum:				-5.0				
Maximum:		3.0	3.0	80.0				
Mass	Calc. Mass	mDa	PPM	DBE	i-FIT	Norm	Conf(%)	Formula
177.1031	177.1028	0.3	1.7	5.5	382.8	n/a	n/a	C10 H13 N2 O

Cone voltage =30V Xevo G2 QTof #YCA210 05-Jul-2022 15:33:15
208F1 74 (0.465) AM2 (Ar,20000.0,0.00,0.00); Cm (70:74-54:64x2.000) 1: TOF MS ES+
1.57e6

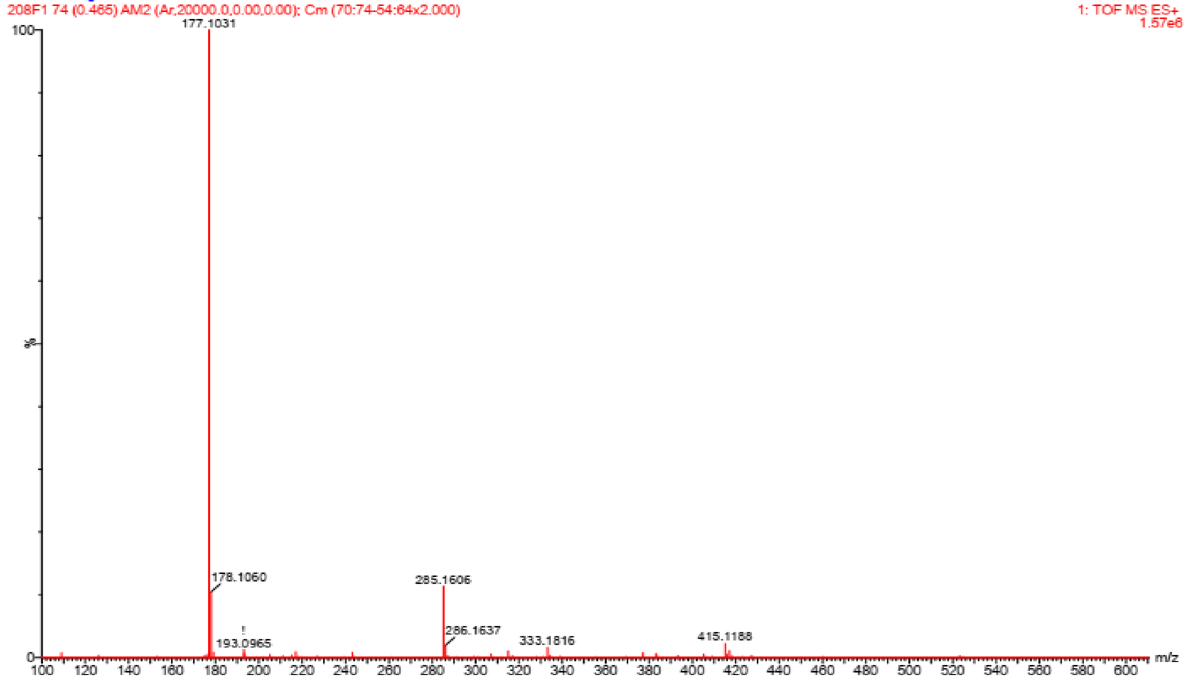

Figure S38. ESI-HRMS spectra of **6d**

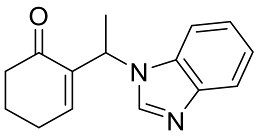

7a

Elemental Composition Report **Page 1**

Single Mass Analysis
Tolerance = 3.0 PPM / DBE: min = -5.0, max = 80.0
Element prediction: Off
Number of isotope peaks used for i-FIT = 3

Monoisotopic Mass, Even Electron Ions
333 formula(e) evaluated with 1 results within limits (up to 50 closest results for each mass)
Elements Used:
C: 0-80 H: 0-100 N: 0-10 O: 0-10

Cone voltage =30V Xevo G2 QTof #YCA210 07-Jul-2022 09:45:00
250F1 68 (0.432) AM2 (Ar,20000.0,0.00,0.00); Cm (63:68-52:62x2.000) 1: TOF MS ES+
2.67e+006

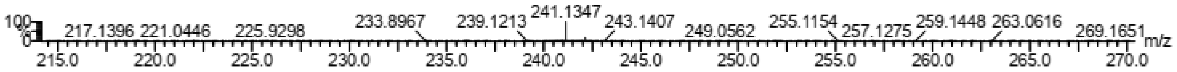

Minimum:				-5.0				
Maximum:		3.0	3.0	80.0				
Mass	Calc. Mass	mDa	PPM	DBE	i-FIT	Norm	Conf(%)	Formula
241.1347	241.1341	0.6	2.5	8.5	537.5	n/a	n/a	C15 H17 N2 O

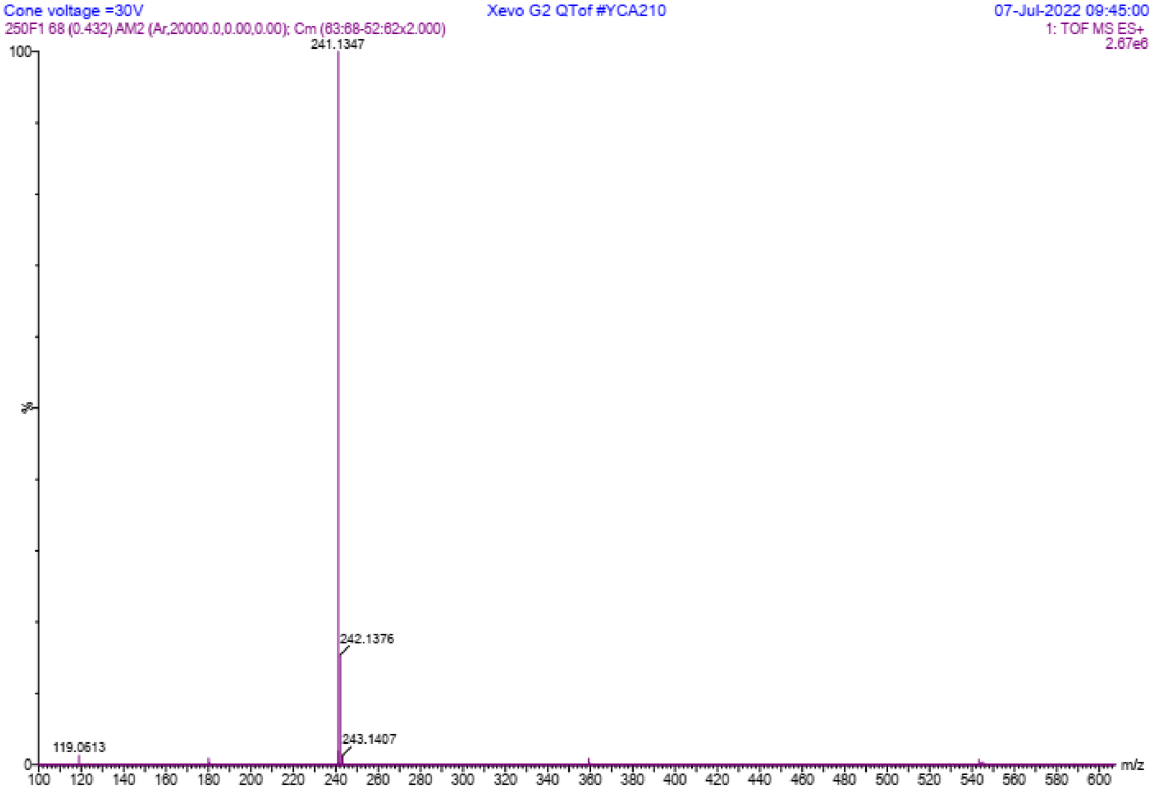

Figure S39. ESI-HRMS spectra of **7a**

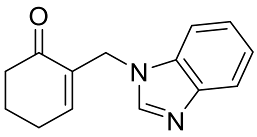

7b

Elemental Composition Report

Page 1

Single Mass Analysis

Tolerance = 3.0 PPM / DBE: min = -5.0, max = 80.0
Element prediction: Off
Number of isotope peaks used for i-FIT = 3

Monoisotopic Mass, Even Electron Ions
307 formula(e) evaluated with 1 results within limits (up to 50 closest results for each mass)
Elements Used:
C: 0-80 H: 0-100 N: 0-10 O: 0-10

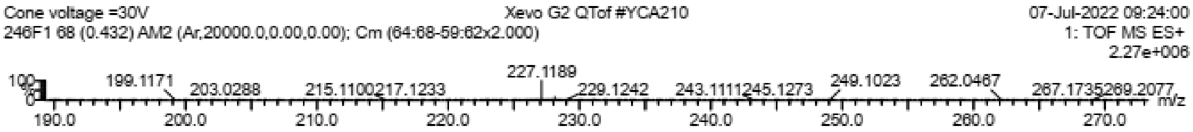

Minimum:				-5.0				
Maximum:		3.0	3.0	80.0				
Mass	Calc. Mass	mDa	PPM	DBE	i-FIT	Norm	Conf(%)	Formula
227.1189	227.1184	0.5	2.2	8.5	530.7	n/a	n/a	C14 H15 N2 O

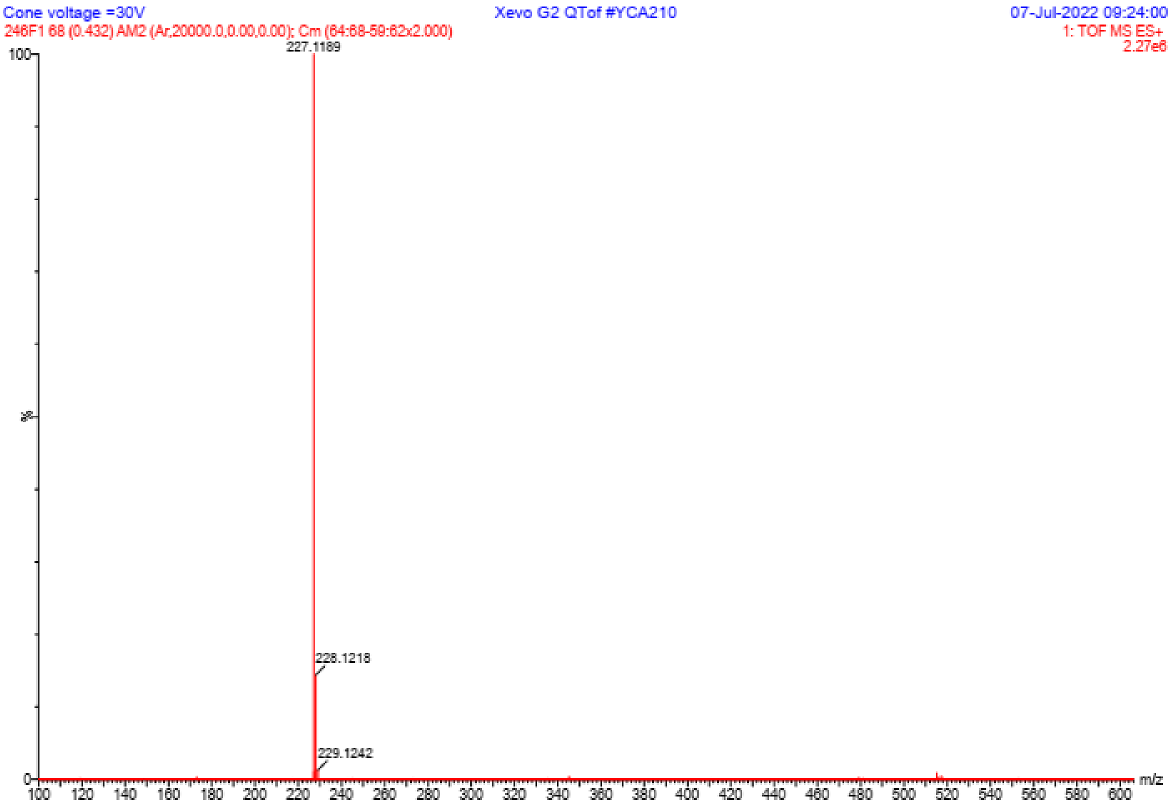

Figure S40. ESI-HRMS spectra of **7b**

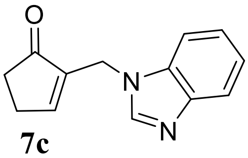

7c

Elemental Composition Report **Page 1**

Single Mass Analysis
Tolerance = 3.0 PPM / DBE: min = -5.0, max = 80.0
Element prediction: Off
Number of isotope peaks used for i-FIT = 3

Monoisotopic Mass, Even Electron Ions
277 formula(e) evaluated with 1 results within limits (up to 50 closest results for each mass)
Elements Used:
C: 0-80 H: 0-100 N: 0-10 O: 0-10

Cone voltage =30V Xevo G2 QTof #YCA210 07-Jul-2022 09:28:03
247F1 68 (0.432) AM2 (Ar,20000.0,0.00,0.00); Cm (65:68-60:65x2.000) 1: TOF MS ES+ 1.28e+006

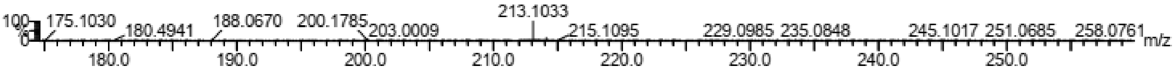

Minimum:				-5.0				
Maximum:		3.0	3.0	80.0				
Mass	Calc. Mass	mDa	PPM	DBE	i-FIT	Norm	Conf(%)	Formula
213.1033	213.1028	0.5	2.3	8.5	414.9	n/a	n/a	C13 H13 N2 O

Cone voltage =30V Xevo G2 QTof #YCA210 07-Jul-2022 09:28:03
247F1 68 (0.432) AM2 (Ar,20000.0,0.00,0.00); Cm (65:68-60:65x2.000) 1: TOF MS ES+ 1.28e6

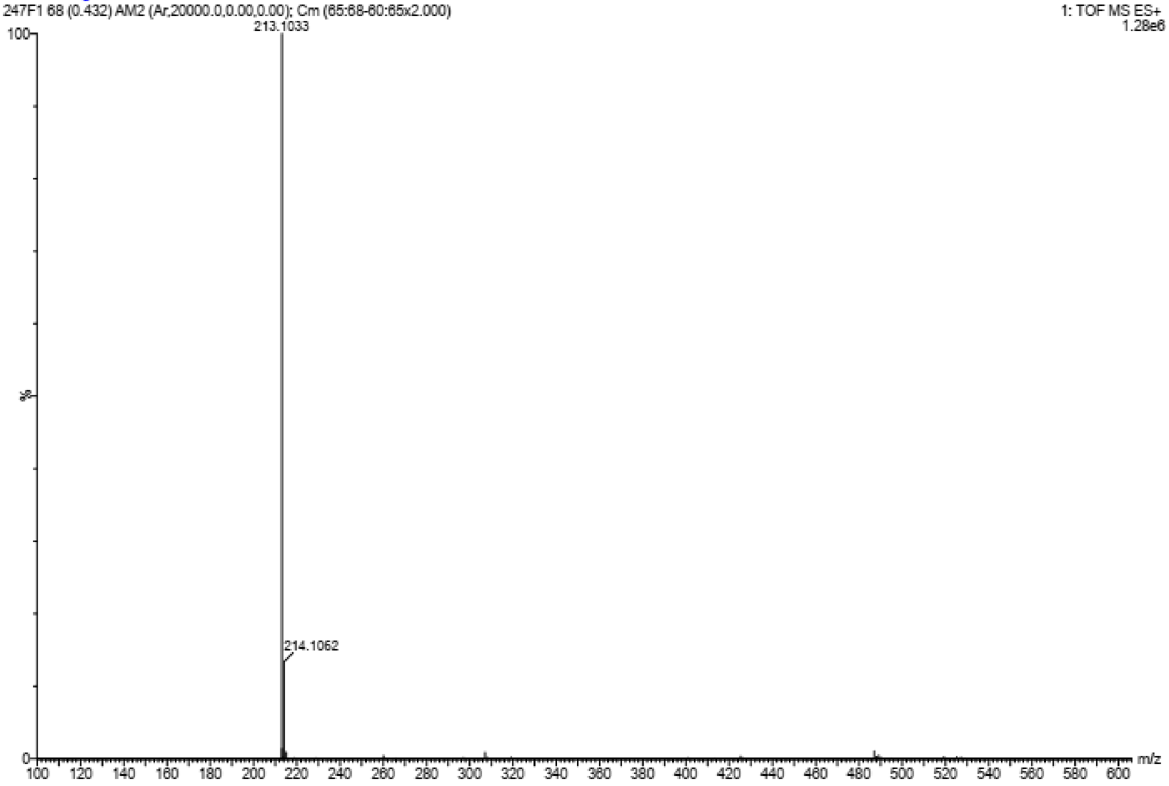

Figure S41. ESI-HRMS spectra of **7c**

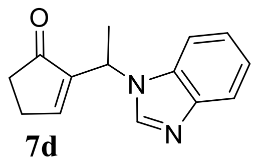

7d

Elemental Composition Report

Page 1

Single Mass Analysis

Tolerance = 3.0 PPM / DBE: min = -5.0, max = 80.0
Element prediction: Off
Number of isotope peaks used for i-FIT = 3

Monoisotopic Mass, Even Electron Ions
307 formula(e) evaluated with 1 results within limits (up to 50 closest results for each mass)
Elements Used:
C: 0-80 H: 0-100 N: 0-10 O: 0-10

Cone voltage =30V Xevo G2 QTof #YCA210 07-Jul-2022 09:40:59
249F2 68 (0.432) AM2 (Ar,20000.0,0.00,0.00); Cm (63:68-58:63x2.000) 1: TOF MS ES+
1.46e+006

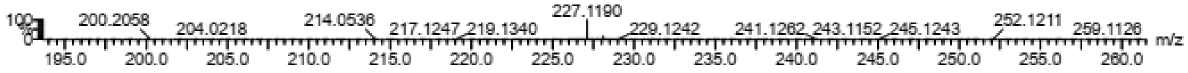

Minimum:				-5.0				
Maximum:		3.0	3.0	80.0				
Mass	Calc. Mass	mDa	PPM	DBE	i-FIT	Norm	Conf(%)	Formula
227.1190	227.1184	0.6	2.6	8.5	502.4	n/a	n/a	C14 H15 N2 O

Cone voltage =30V Xevo G2 QTof #YCA210 07-Jul-2022 09:40:59
249F2 68 (0.432) AM2 (Ar,20000.0,0.00,0.00); Cm (63:68-58:63x2.000) 1: TOF MS ES+
1.46e6

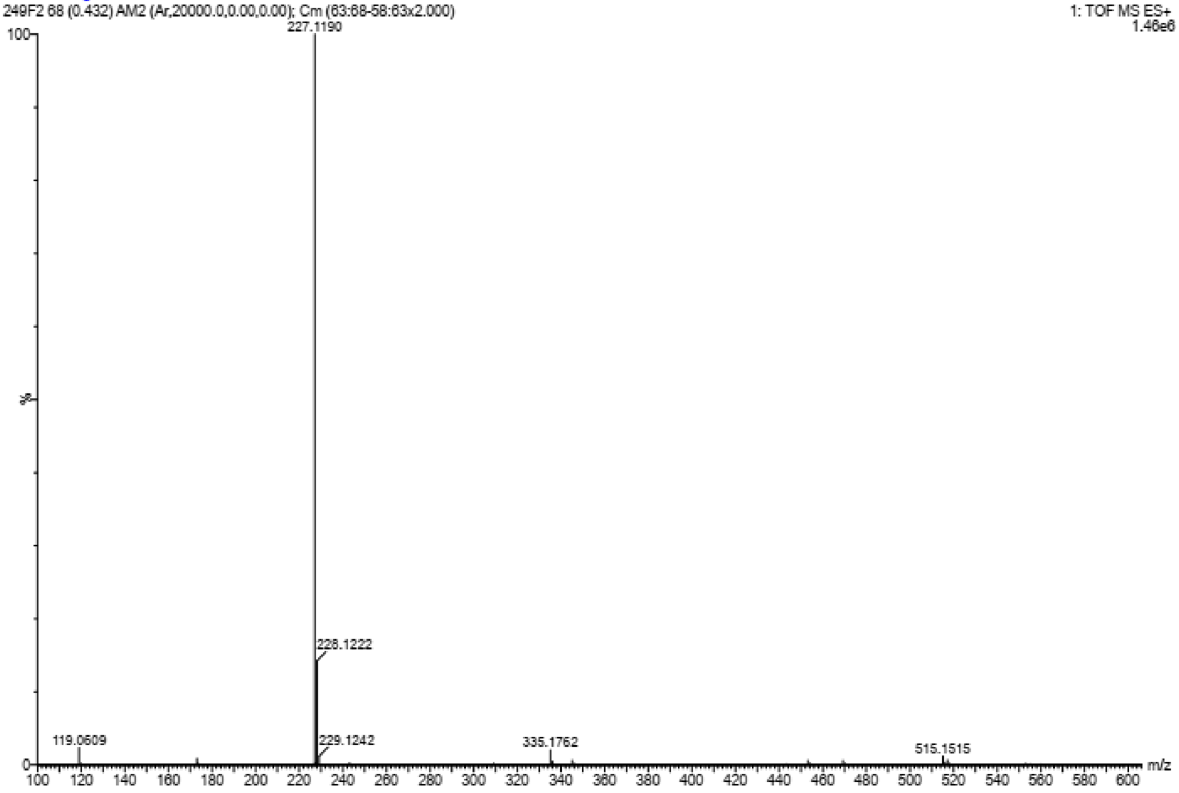

Figure S42. ESI-HRMS spectra of **7d**

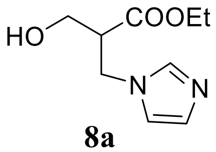

8a

Elemental Composition Report

Page 1

Single Mass Analysis

Tolerance = 3.0 PPM / DBE: min = -5.0, max = 80.0
Element prediction: Off
Number of isotope peaks used for i-FIT = 3

Monoisotopic Mass, Even Electron Ions
249 formula(e) evaluated with 1 results within limits (up to 50 closest results for each mass)
Elements Used:
C: 0-80 H: 0-100 N: 0-10 O: 0-10

Cone voltage =30V Xevo G2 QTof #YCA210 05-Jul-2022 15:38:31
252 76 (0.476) AM2 (Ar,20000.0,0.00,0.00); Cm (72:76-56:65x2.000) 1: TOF MS ES+ 2.03e+006

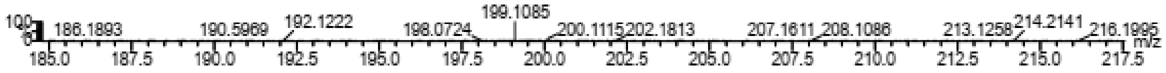

Minimum:				-5.0				
Maximum:		3.0	3.0	80.0				
Mass	Calc. Mass	mDa	PPM	DBE	i-FIT	Norm	Conf(%)	Formula
199.1085	199.1083	0.2	1.0	3.5	466.3	n/a	n/a	C9 H15 N2 O3

Cone voltage =30V Xevo G2 QTof #YCA210 05-Jul-2022 15:38:31
252 76 (0.476) AM2 (Ar,20000.0,0.00,0.00); Cm (72:76-56:65x2.000) 1: TOF MS ES+ 2.03e6

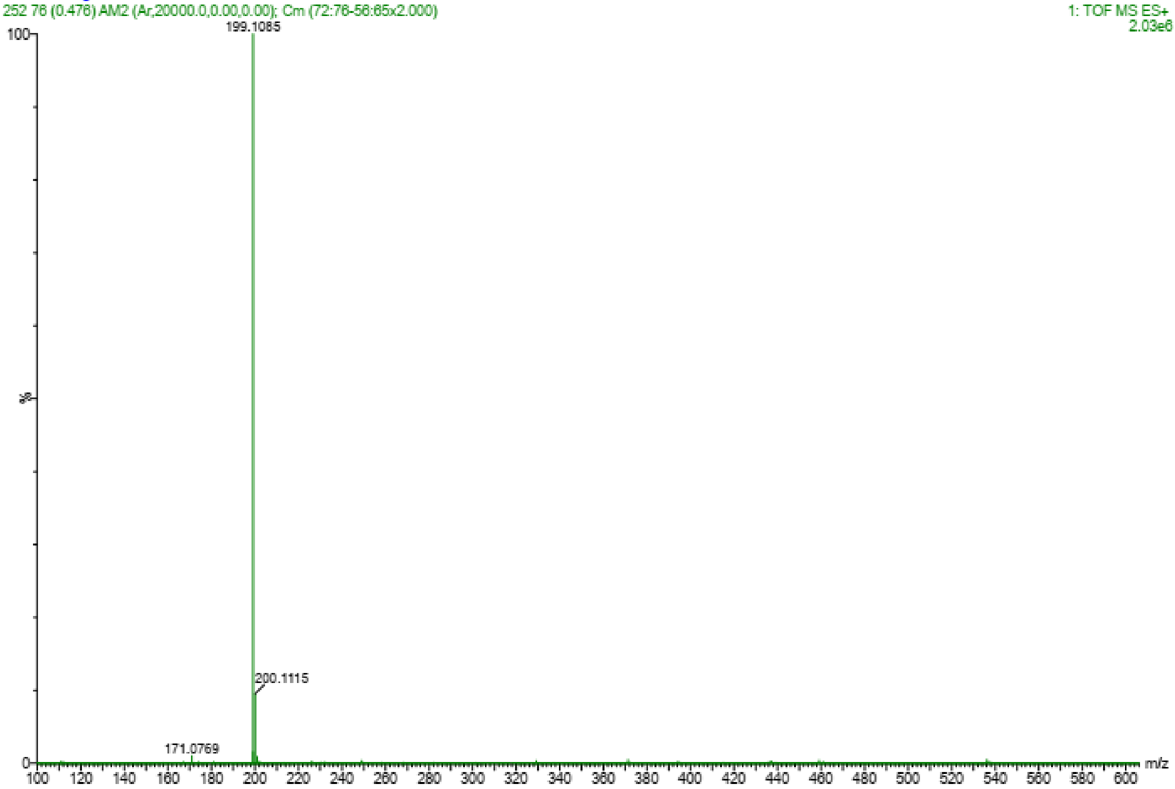

Figure S43. ESI-HRMS spectra of **8a**

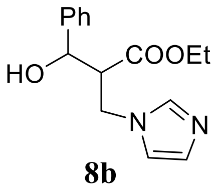

8b

Elemental Composition Report

Page 1

Single Mass Analysis
Tolerance = 3.0 PPM / DBE: min = -5.0, max = 80.0
Element prediction: Off
Number of isotope peaks used for i-FIT = 3

Monoisotopic Mass, Even Electron Ions
406 formula(e) evaluated with 1 results within limits (up to 50 closest results for each mass)
Elements Used:
C: 0-80 H: 0-100 N: 0-10 O: 0-10

Cone voltage =30V
Xevo G2 QTof #YCA210
07-Jul-2022 09:57:08
255F0 70 (0.443) AM2 (Ar,20000.0,0.00,0.00); Cm (63:70-61:64x2.000)
1: TOF MS ES+
3.76e+006

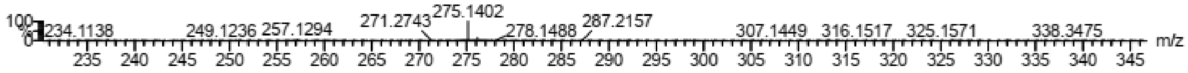

Mass	Calc. Mass	mDa	PPM	DBE	i-FIT	Norm	Conf(%)	Formula
Minimum:				-5.0				
Maximum:		3.0	3.0	80.0				
275.1402	275.1396	0.6	2.2	7.5	599.2	n/a	n/a	C15 H19 N2 O3

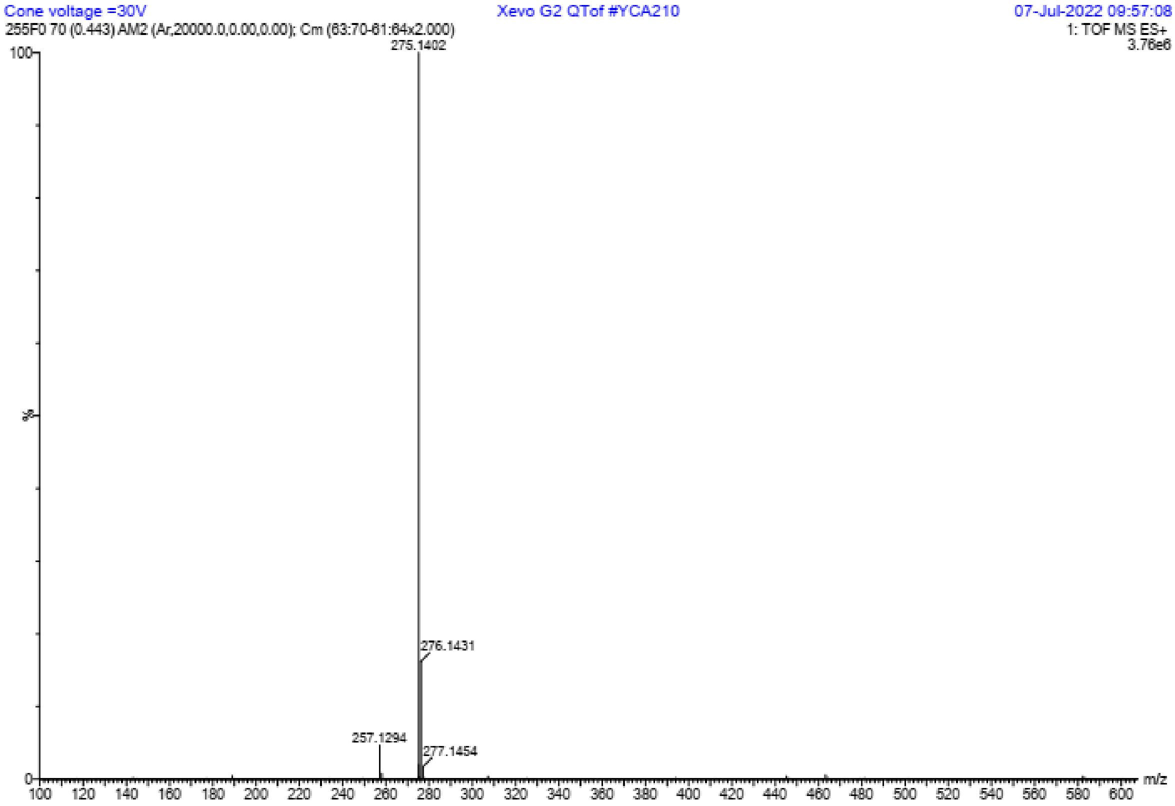

Figure S44. ESI-HRMS spectra of **8b**

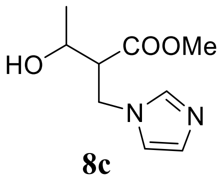

8c

Elemental Composition Report

Page 1

Single Mass Analysis

Tolerance = 3.0 PPM / DBE: min = -5.0, max = 80.0
Element prediction: Off
Number of isotope peaks used for i-FIT = 3

Monoisotopic Mass, Even Electron Ions
249 formula(e) evaluated with 1 results within limits (up to 50 closest results for each mass)
Elements Used:
C: 0-80 H: 0-100 N: 0-10 O: 0-10

Cone voltage =30V | Xevo G2 QTof #YCA210 | 07-Jul-2022 10:01:13
256F2 72 (0.454) AM2 (Ar,20000.0,0.00,0.00); Cm (68:72-58:62x2.000)
1: TOF MS ES+
4.37e+005

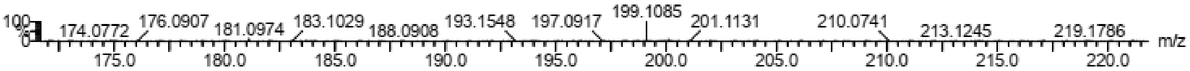

Minimum:				-5.0				
Maximum:		3.0	3.0	80.0				
Mass	Calc. Mass	mDa	PPM	DBE	i-FIT	Norm	Conf(%)	Formula
199.1085	199.1083	0.2	1.0	3.5	293.4	n/a	n/a	C9 H15 N2 O3

Cone voltage =30V | Xevo G2 QTof #YCA210 | 07-Jul-2022 10:01:13
256F2 72 (0.454) AM2 (Ar,20000.0,0.00,0.00); Cm (68:72-58:62x2.000)
1: TOF MS ES+
4.37e5

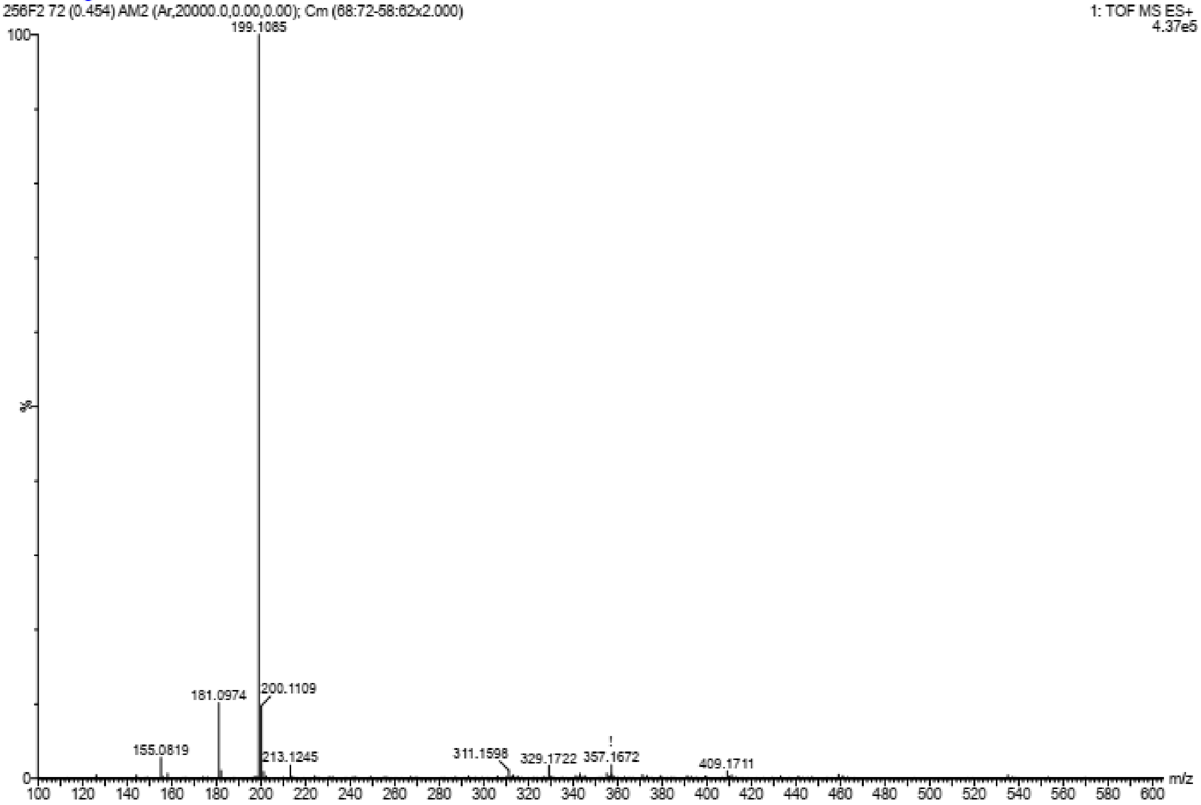

Figure S45. ESI-HRMS spectra of **8c**

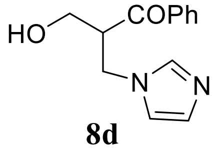

8d

Elemental Composition Report **Page 1**

Single Mass Analysis
Tolerance = 5.0 PPM / DBE: min = -1.5, max = 80.0
Element prediction: Off

Monoisotopic Mass, Odd and Even Electron Ions
111 formula(e) evaluated with 1 results within limits (all results (up to 1000) for each mass)
Elements Used:
C: 0-100 H: 0-100 N: 0-5 O: 0-5

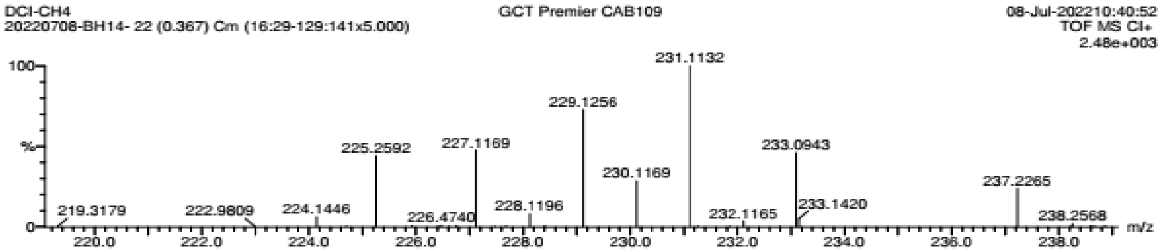

Minimum:				-1.5		
Maximum:		1.5	5.0	80.0		
Mass	Calc. Mass	mDa	PPM	DBE	i-FIT	Formula
231.1132	231.1134	-0.2	-0.9	7.5	327.2	C13 H15 N2 O2

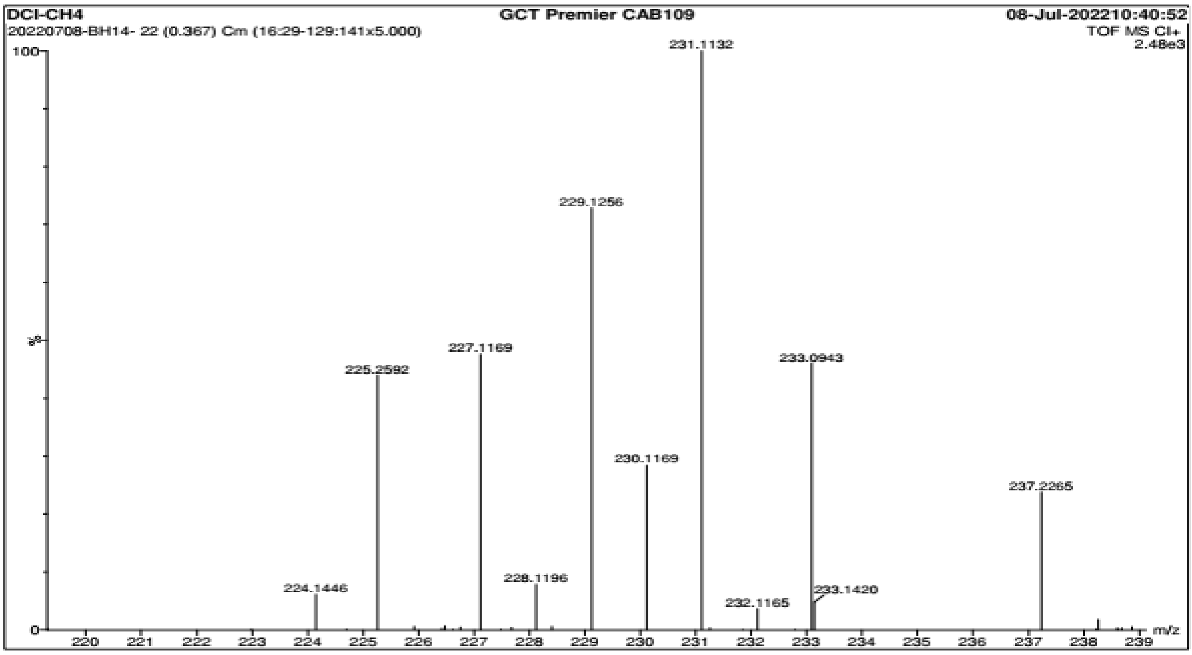

Figure S46. ESI-HRMS spectra of **8d**

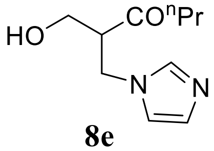

8e

Elemental Composition Report

Page 1

Single Mass Analysis

Tolerance = 3.0 PPM / DBE: min = -5.0, max = 80.0
Element prediction: Off
Number of isotope peaks used for i-FIT = 3

Monoisotopic Mass, Even Electron Ions
243 formula(e) evaluated with 1 results within limits (up to 50 closest results for each mass)
Elements Used:
C: 0-80 H: 0-100 N: 0-10 O: 0-10

Cone voltage =15V Xevo G2 QTof #YCA210 07-Jul-2022 10:13:19
BH19 83 (0.529) AM2 (Ar,20000.0,0.00,0.00); Cm (83:86-42:54x2.000) 2: TOF MS ES+
2.58e+006

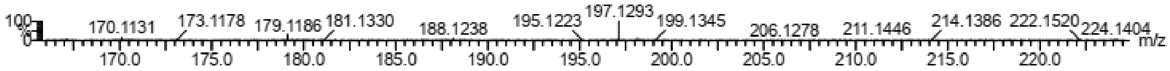

Mass	Calc. Mass	mDa	PPM	DBE	i-FIT	Norm	Conf(%)	Formula
Minimum:				-5.0				
Maximum:		3.0	3.0	80.0				
197.1293	197.1290	0.3	1.5	3.5	588.4	n/a	n/a	C10 H17 N2 O2

Cone voltage =15V Xevo G2 QTof #YCA210 07-Jul-2022 10:13:19
BH19 83 (0.529) AM2 (Ar,20000.0,0.00,0.00); Cm (83:86-42:54x2.000) 2: TOF MS ES+
2.58e6

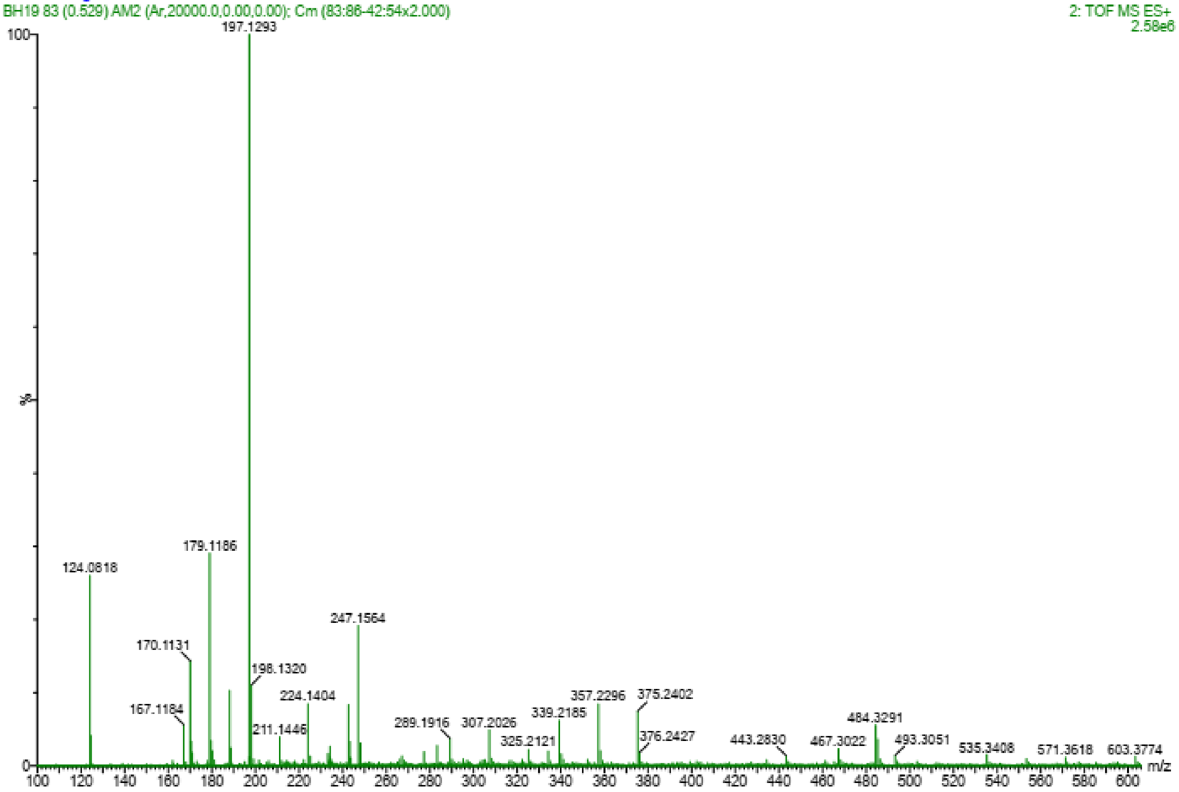

Figure S47. ESI-HRMS spectra of **8e**

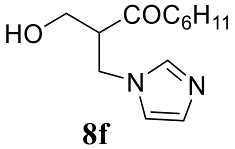

8f

Elemental Composition Report

Page 1

Single Mass Analysis

Tolerance = 3.0 PPM / DBE: min = -5.0, max = 80.0
Element prediction: Off
Number of isotope peaks used for i-FIT = 3

Monoisotopic Mass, Even Electron Ions
320 formula(e) evaluated with 1 results within limits (up to 50 closest results for each mass)
Elements Used:
C: 0-80 H: 0-100 N: 0-10 O: 0-10

Cone voltage =15V Xevo G2 QTof #YCA210 05-Jul-2022 15:42:36
BH16 83 (0.528) AM2 (Ar,20000.0,0.00,0.00); Cm (81:94-61:67x2.000) 2: TOF MS ES+
7.24e+006

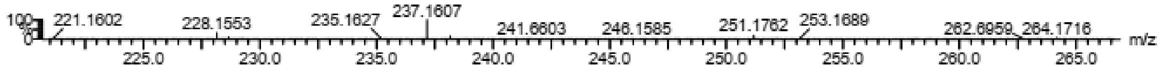

Minimum:				-5.0				
Maximum:		3.0	3.0	80.0				
Mass	Calc. Mass	mDa	PPM	DBE	i-FIT	Norm	Conf(%)	Formula
237.1607	237.1603	0.4	1.7	4.5	687.8	n/a	n/a	C13 H21 N2 O2

Cone voltage =15V Xevo G2 QTof #YCA210 05-Jul-2022 15:42:36
BH16 83 (0.528) AM2 (Ar,20000.0,0.00,0.00); Cm (81:94-61:67x2.000) 2: TOF MS ES+
7.24e6

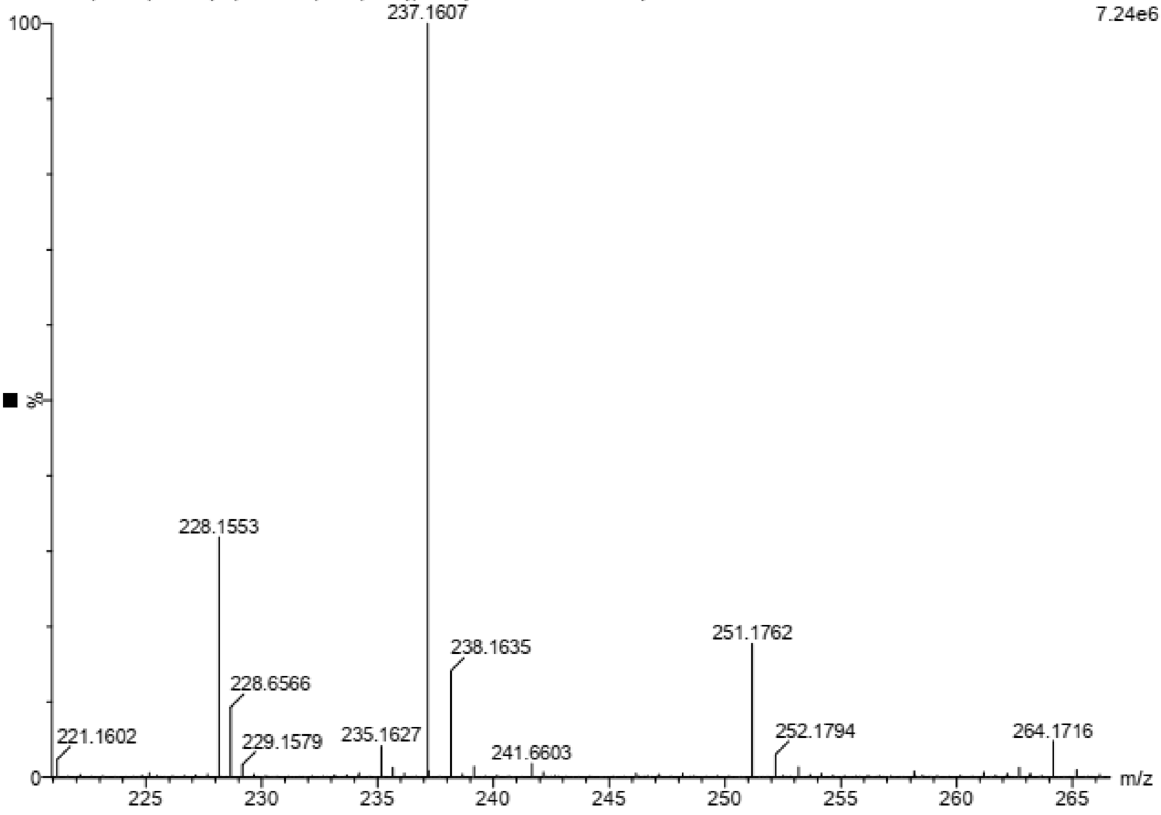

Figure S48. ESI-HRMS spectra of **8f**
